# Laccase-Catalyzed Oxidation of Allylbenzene Derivatives: Towards a Green Equivalent of Ozonolysis

**DOI:** 10.3390/molecules26196053

**Published:** 2021-10-06

**Authors:** Mathilde Lecourt, Giorgiana Chietera, Bernard Blerot, Sylvain Antoniotti

**Affiliations:** 1 Institut de Chimie de Nice, Université Côte d’Azur, CNRS, Parc Valrose, CEDEX 2, 06108 Nice, France; mathilde.lecourt@univ-cotedazur.fr; 2 LMR Naturals by IFF, Parc d’Activité les Bois de Grasse, 18 Avenue Joseph Honoré Isnard, 06130 Grasse, France; Giorgiana.CHIETERA@IFF.com (G.C.); bernard.blerot@iff.com (B.B.)

**Keywords:** biocatalysis, sustainability, phenylpropanoids

## Abstract

Laccase-based biocatalytic reactions have been tested with and without mediators and optimized in the oxidation of allylbenzene derivatives, such as methyl eugenol taken as a model substrate. The reaction primarily consisted in the hydroxylation of the propenyl side chain, either upon isomerization of the double bond or not. Two pathways were then observed; oxidation of both allylic alcohol intermediates could either lead to the corresponding α,β-unsaturated carbonyl compound, or the corresponding benzaldehyde derivative by oxidative cleavage. Such a process constitutes a green equivalent of ozonolysis or other dangerous or waste-generating oxidation reactions. The conversion rate was sensitive to the substitution patterns of the benzenic ring and subsequent electronic effects.

## 1. Introduction

Oxidation processes in organic chemistry are typically waste-generating processes [1,2]. Not only that the fundamental principles of oxidation of organic substrates require a stoichiometric electron acceptor, but that suitable redox potentials to make these transformations efficient have led to the massive use of metallic oxides eventually leaving metallic by-products. In order to mitigate this undesired effect, combinations of catalytic systems with benign terminal oxidants, such as molecular oxygen [3], hydrogen hydroperoxide [4] or bleach [5], have been developed. This is what Nature does for millions of years with enzymes evolved to achieve specific, selective, and efficient oxidation reactions of organic substrates using indeed O_2_ or H_2_O_2_ to activate metal centers (mostly Cu, Fe, Mn…) within their active sites. It is thus tempting to use these enzymes to perform oxidation reactions at laboratory or pilot and full-industrial scale in biocatalytic processes [6,7].

Biocatalysis is a branch of catalysis involving enzymes or cells as catalysts for a given reaction. Biocatalytic processes tolerate mild operating conditions (temperature, pH, pressure) and can be run in aqueous mediums as far as the substrate has sufficient solubility [8]. Enzymes can also be directly used in vivo by applying wild or engineered cells to the substrate [9,10]. Besides that, enzymes display selectivity and/or specificity that are of great interest either in fine chemical synthesis or in pharmaceutical or perfumery areas [9,11,12,13]. Engineered enzymes are more and more often used in industry to replace late steps in a chemical synthesis where high degrees of selectivity and efficiency are required and/or to improve the processes’ sustainability [14,15,16].

Laccases belong to the large oxidoreductase class. This family of enzymes can genuinely convert a wide range of compounds, which can be broadened by using mediators for oxidation transfer [14,15,17,18]. Laccases were investigated on various substrates such as phenols, methoxybenzenes, alkenes and polycyclic aromatic hydrocarbons [19,20,21,22]. These enzymes are employed for lignin degradation in Kraft pulp [23,24,25] and in phenol depolluting processes [26,27] and are broadly considered in green oxidation catalytic systems [14,15,28].

Methyl eugenol **1** is an allylbenzene derivative naturally produced by plants. It was identified as a product of lignin degradation in the presence of laccase from *Trametes versicolor* and methyl syringate as natural mediators [29]. As **1** is an allergen and a carcinogenic agent [30,31,32]. It is strongly regulated in food and cosmetics [33,34,35]. Its metabolization by microorganisms involves cytochrome P450, which is an oxidase [36,37]. Its antifungal and cytotoxic effects were also investigated [38,39].

**Figure 1:**
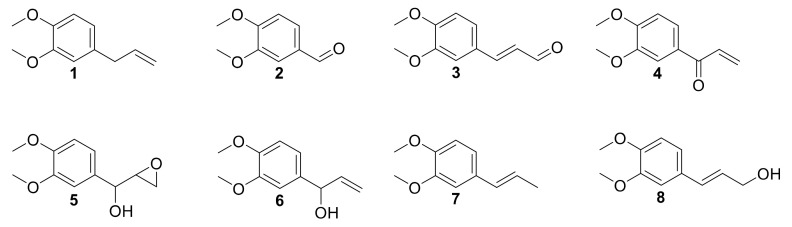
Methyl eugenol **1** and derivatives.

Laccase has been already investigated in the oxidation of phenols, such as eugenol and isoeugenol, and was shown to induce oxidative dimerization [40,41]. To our knowledge, laccase-catalyzed oxidation of **1** and allylbenzene derivatives has never been studied. Herein, we investigated the activity of laccases on allylbenzene derivatives, including **1,** in a quest for alternative sustainable oxidative protocols. Intermediates and products were thus isolated, identified and engaged separately in order to better understand the mechanism of the reaction (Figure 1).

## 2. Materials and Methods

*Chemicals*: Chemicals, organic solvents and laccase from *Trametes versicolor* were purchased from Sigma-Aldrich (Saint-Louis, MO, USA) and used as received. Distilled water was obtained by filtration on an Elix^®^ Reference 3 system with a Progard^®^S2 cartridge. Laccase M120 was obtained as a kind loan from Amano Enzyme Inc. (United Kingdom), and laccase Mrs was provided by ISM2 Laboratory, France (Pierre Rousselot Pailley, Thierry Tron, Equipe Biosciences), obtained after heterologous expression and purification. Both were stored at −18 °C.

*Analysis*: ^1^H and ^13^C NMR spectra were recorded on Bruker Avance 400 spectrometer. Chemical shifts, reported in ppm, were referenced relatively to TMS on the signals of residual solvents (^1^H and ^13^C). GC/MS analyses were carried out with an Agilent 7820A chromatograph coupled to an Agilent 5977B MS detector (Agilent, California). Samples were analyzed on a capillary column SAPIENS-5MS (10 m × 0.10 mm × 0.10 µm, 5% diphenyl-95% dimethylpolysiloxane, Teknokroma, Spain). Operating conditions: carrier gas: He, constant flow, 20 mL/min; injection temp., 250 °C, injection volume, 0.2 µL, split ratio, 1:50; temp. program: 50 °C held 1 min, 50 °C to 280 °C at 10 °C/min, then held isothermal (10 min), ion source temp., 230 °C; transfer line temp., 300 °C; ionization energy, 70 eV; electron ionization mass spectra were acquired over the mass range of 35–550 amu.

Identification of the products was based on comparison of their MS spectra with those of commercial libraries (NIST15, Wiley), laboratory MS libraries built up from pure substances, with MS literature data, or comparison with authentic samples.

*Standard procedure for the enzymatic oxidation of allylbenzene derivatives*: Substrate (1 mmol, 0.25 M), mediator (10 mol%) and laccase (5 wt%) were introduced in a 20 mL vial in a pH5 20 mM aqueous acetate buffer (4 mL). The reaction medium was incubated under an oxygen atmosphere using an incubator shaker with orbital agitation (24 h, 40 °C, 200 rpm). The work-up consisted of EtOAc extraction of the aqueous phase (5 × 2 mL). Organic phases were pooled, dried over MgSO_4_ and concentrated in vacuo. GC/MS and ^1^H-NMR were performed to determine the reaction outcome on crude products during optimization phases and on purified material after column chromatography for synthetic purposes.

*Oxidation intermediates characterization*: Enzymatic oxidation was carried out on a larger scale. ME **1** (11.96 mmol, 0.1 M), HOBt (1.23 mmol, 166.8 mg) and laccase (46 mg) were dissolved in a pH5 20 mM aqueous acetate buffer (120 mL). The mixture was stirred under an oxygen atmosphere in an oil bath at 40 °C. After 24 h, 1 mL of the mixture was submitted to the above-described work-up, and the reaction progress was estimated by GC/MS analysis. In the case of partial conversion, an addition of laccase and mediator was performed (up to 8 additions for a total of 1292.3 mg of HOBt and 349.3 mg of laccase). Upon the completion of the reaction with full conversion, the work-up was performed as described. Different products were isolated by column chromatography on silica gel (diethyl ether/petroleum ether gradient as the eluent).

Compounds **1**, **2** and **7** were purchased from Sigma-Aldrich (Saint-Louis, MO, USA), and compound **3** was purchased from Enamine (Ukraine).

(3,4-Dimethoxyphenyl)prop-2-en-1-one **4**: **6** (2.61 mmol) was diluted in EtOAc (0.12 M). Manganese oxide (MnO_2_, 4.16 eq.) was added, and the mixture was vigorously stirred at room temperature. The conversion was checked by TLC and pushed up with manganese oxide supplementation (8.32 eq.). After 48 h, the black suspension was filtered on a Celite^®^ pad and on silica gel before concentration in vacuo.

^1^H NMR (400 MHz, CDCl_3_) δ (ppm) 7.61–7.56 (m, 2H, H_Ar_), 7.19 (dd, *J* = 17.0, 10.5 Hz, 1H, H_1_), 6.90 (d, *J* = 8.4 Hz, 1H, H_Ar_), 6.43 (dd, *J* = 17.0, 1.8 Hz, 1H, H_3_), 5.87 (dd, *J* = 10.5, 1.8 Hz, 1H, H_3_), 3.96 and3.95 (s, 3H, OCH_3_).

^13^C NMR (100 MHz, CDCl_3_) δ (ppm) 189.28, (C=O); 153.57 and 149.40 (*C*Ar-OCH_3_); 132.07, (CH=); 130.56, (CAr); 129.39, (=CH_2_); 123.56, (CHAr); 110.92, (CHAr); 110.11, (CHAr); 56.23, (OCH_3_); 56.15, (OCH_3_).

MS m/z (%): 192 (M^+•^, 50), 165 (100), 122 (20), 107 (22), 91 (20), 79 (78), 77 (40), 63 (25), 55 (60) and 51 (39).

1-(3,4-Dimethoxyphenyl)-2,3-epoxypropan-1-ol **5**: Isolated as a mixture of diastereomers from the reaction of **1**.

^1^H NMR (400 MHz, CDCl_3_): 3:7 mixture of 2 diastereomers. δ (ppm) 6.99–6.85 (m, 3H, H_Ar_), 4.87 and 4.44 (d, 1H, H_1_), 3.91 and 3.81 (s, 3H, OCH_3_), 3.23 (m, 1H, H_2_), 2.77–2.97 (m, 2H, H_3_).

^13^C NMR (100 MHz, CDCl_3_): 3:7 mixture of 2 diastereomers. δ (ppm) 149.36 and 149.12 (*C*Ar-OCH_3_); 149.31 and 149.16 (C’Ar-OCH_3_); 132.99, (CAr); 132.09, (C’Ar); 118.92, (C’HAr); 118.81, (CHAr); 111.25, (CHAr); 109.68, (C’HAr); 109.58, (CHAr); 109.55, (C’HAr); 74.27, (C-OH); 70.84, (C’-OH); 56.09–56.05 and 56.02, (CH, OCH_3_, OCH_3_); 56.09–56.05 and 55.17, (C’H, OC’H_3_, OC’H_3_); 45.50, (CH_2_); 43.79, (C’H_2_).

MS *m*/*z* (%): diastereomer 1 (maj.) 210 (M^+•^,35), 167 (100), 165 (14), 151 (54), 139 (67), 124 (23), 108 (19), 107 (15), 77 (25) and 65 (15).

1-(3,4-Dimethoxyphenyl)prop-2-en-1-ol **6**: **2** (8.86 mmol) under a nitrogen atmosphere was dissolved in distilled THF (0.11 M) and put in a −80 °C bath. Vinyl magnesium bromide (C_2_H_3_MgBr, 1.19 eq.) in THF was added slowly. The reaction was stirred and let warm to room temperature until TLC analysis indicated that no starting material remained (7 h). The reaction was quenched with a saturated aqueous NaCl solution and extracted with EtOAc. The combined organic layers were washed with an aqueous solution of saturated NaCl, dried on MgSO_4_, filtrated and concentrated in vacuo (81%).

^1^H NMR (400 MHz, CDCl_3_) δ (ppm) 6.89–6.85 (m, 2H, H_Ar_); 6.81 (m, 1H, H_Ar_), 6.02 (ddd, *J* = 17.2, 10.3, 5.9 Hz, 1H, H_2_), 5.33–5.15 (m, 2H, H_3_), 5.11 (dd, *J* = 5.9, 1.5 Hz, 1H, H_1_), 3.85 and 3.84 (s, 3H, OCH_3_). ^13^C NMR (100 MHz, CDCl_3_) δ (ppm) 149.21 and 148.71 (*C*Ar-OCH_3_); 140.37, (CH=); 135.41, (CAr); 118.76, (CHAr); 115.01, (=CH_2_); 111.13, (CHAr); 109.59, (CHAr); 75.18, (CH-OH); 56.04, (OCH_3_); 55.95, (OCH_3_).

MS m/z (%): 194 (M^+•^, 97), 165 (29), 163 (62), 151 (33), 139 (100), 138 (27), 91 (34), 79 (31), 77 (36) and 55 (51).

3-(3,4-Dimethoxyphenyl)prop-2-en-1-ol **8**: **3** (1 mmol) was dissolved in EtOH (0.35 M) and placed under a nitrogen atmosphere. NaBH_4_ (1.25 eq.) was added. The mixture was then stirred for 1.2 h at room temperature. The work-up consisted of EtOH evaporation under vacuo, solubilization in distilled water and extraction with EtOAc. Organic layers were pooled, dried over MgSO_4_ filtrated and concentrated in vacuo (100%).

^1^H NMR (400 MHz, CDCl_3_): δ (ppm) 6.98–6.88 (m, 2H, H_Ar_), 6.82 (d, *J* = 8.2 Hz, 1H, H_Ar_), 6.55 (d, *J* = 15.9 Hz, 1H, H_3_), 6.25 (dt, *J* = 15.9, 5.8 Hz, 1H, H_2_), 4.31 (d, *J* = 5.8 Hz, 2H, H_1_), 3.90 and 3.88 (s, 3H, OCH_3_).

^13^C NMR (100 MHz, CDCl_3_): δ (ppm) 149.15 and 149.04 (*C*Ar-OCH_3_), 131.27 (CAr), 129.86 (CH=), 126.67 (=CH), 119.81 (CHAr), 111.24 (CHAr), 108.97 (CHAr), 63.95 (CH_2_), 56.04 (OCH_3_) and 55.94 (OCH_3_).

MS m/z (%): 194 (M^+•^, 68), 165 (14), 151 (100), 138 (53), 119 (16), 107 (13), 91 (33), 79 (14), 77 (26) and 55 (13).

## 3. Results and Discussion

### 3.1. Enzymatic Reaction of Methyleugenol

To study the reaction, **1** was taken as the model substrate for preliminary investigations. Parameters of **1** oxidation by laccase were screened with a focus on enzyme weight ratio, mediator nature and ratio, co-solvent, pH and temperature.

#### 3.1.1. Controls

A couple of negative controls were performed. For the first control, **1** (1.03 mmol, 185.0 mg) and laccase (10% LA, 19.10 mg) were incubated in aqueous acetate buffer pH5 under an oxygen atmosphere for 24 h at 40 °C, before extraction with EtOAc. ME **1** was quantitively recovered unchanged, with no conversion being noticed by GC/MS analysis. For the second control performed with two mediators, **1** (1.03 mmol, 184.2 mg and 1.02 mmol, 182.2 mg) and mediator (20% HOBt, 0.20 mmol, 27.3 mg and 20% TEMPO, 0.23 mmol, 35.5 mg) were incubated in aqueous acetate buffer pH5 under an oxygen atmosphere for 24 h at 40 °C before extraction with EtOAc. ME **1** was quantitively recovered unchanged, with no conversion being noticed by GC/MS neither. It can be concluded from these controls that laccase is not able to oxidize **1** on its own and that the mediator alone cannot oxidize **1** either. Both mediator and laccase are involved in the oxidation mechanism.

#### 3.1.2. Effect of pH and Temperature

**Figure 2:**
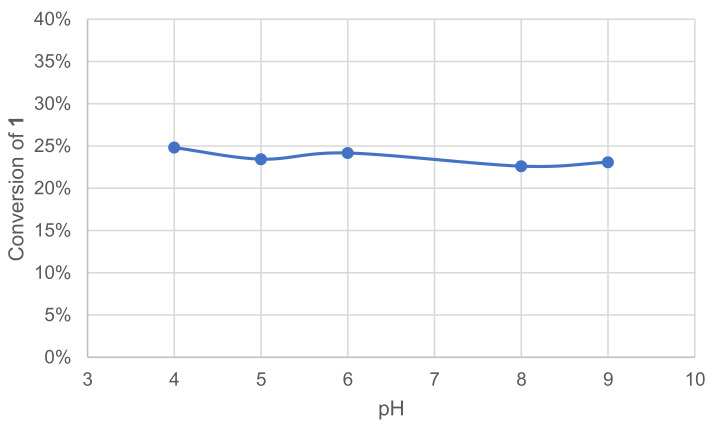
Effect of pH on the conversion of **1** in the laccase-catalyzed oxidation (LA 10%*_w/w_*, HOBt 20 mol%, buffer 0.25 M, O_2_ atmosphere, incubation for 24 h, 40 °C, 200 rpm).

Laccases are known to have optimal pH ranges between 3.5 and 5 with hydrogen donor substrates [15]. To specifically evaluate the effect of pH on our reactions of interest, five different pH were tested (4–9), along with an additional test in deionized water (DIW). The effect was quickly assessed by monitoring the remaining **1** in the crude reaction mixture after extraction and GC/MS analysis (Figure 2).

Our experiments show the stability of laccase–mediator oxidation of **1** in the 4–9 pH range and in DIW (not shown).

**Figure 3:**
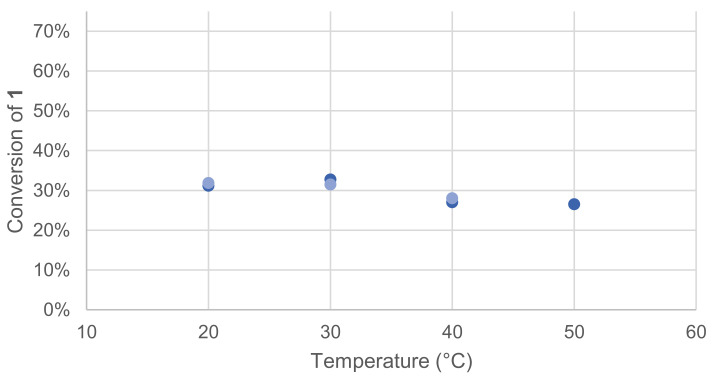
Effect of temperature on the conversion of **1** in the laccase-catalyzed oxidation (LA 10%*_w/w_*, HOBt 20 mol%, acetate buffer pH5 0.25 M, O_2_ atmosphere, incubation for 24 h, 200 rpm). Reactions run in duplicate.

Laccases optimal temperatures are known to be in the range from 50 to 70 °C, but they can also exhibit activity at a lower temperature, such as physiological temperature [15]. To specifically evaluate the effect of temperature on our model reaction, four different temperatures were tested. Once again, the effect was quickly assessed by monitoring the remaining **1** in the crude reaction mixture after extraction and GC/MS analysis (Figure 3).

The optimal temperature was found to be between 20 and 30 °C for the reaction of **1**.

#### 3.1.3. Effect of Co-Solvent

**Figure 4:**
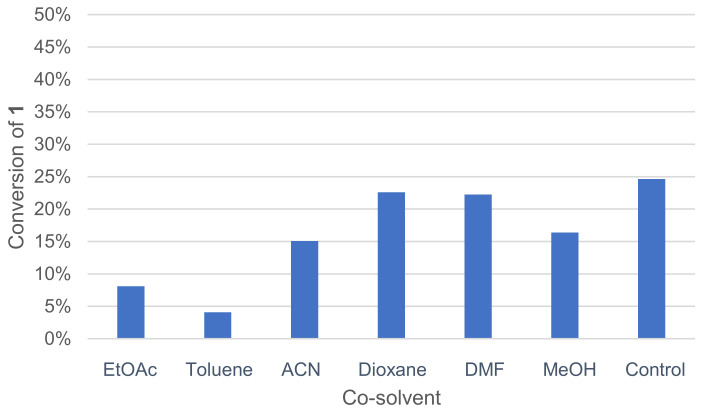
Effect of co-solvents on the conversion of **1** in the laccase-catalyzed oxidation (LA 10%*_w/w_*, HOBt 20 mol%, acetate buffer pH5 0.25 M, co-solvent 50%*_v/v_* _buffer_, O_2_ atmosphere, incubation for 24 h, 40 °C, 200 rpm).

A co-solvent can be added to the medium to improve substrate solubility in water. The positive effect of co-solvent such as EtOAc on 2,6-dimethoxyphenol dimerization by *Trametes pubescens* laccase was already reported [42]. Various co-solvents, miscible or not with water, were tested, and the activity was measured by GC/MS analysis (Figure 4).

Toluene and EtOAc, which are immiscible with water at this temperature, gave the worst conversion rates. DMF and dioxane allowed for higher conversion rates.

#### 3.1.4. Effect of Oxygenation of the Medium

Medium oxygenation can be a limiting factor of an aerobic oxidation reaction as the oxygen dissolution rate in water is low. An oxygen solubility of 39.04 mg/L in distilled water at 20 °C has been determined for a saturated O_2_ atmosphere [43].

**Figure 5:**
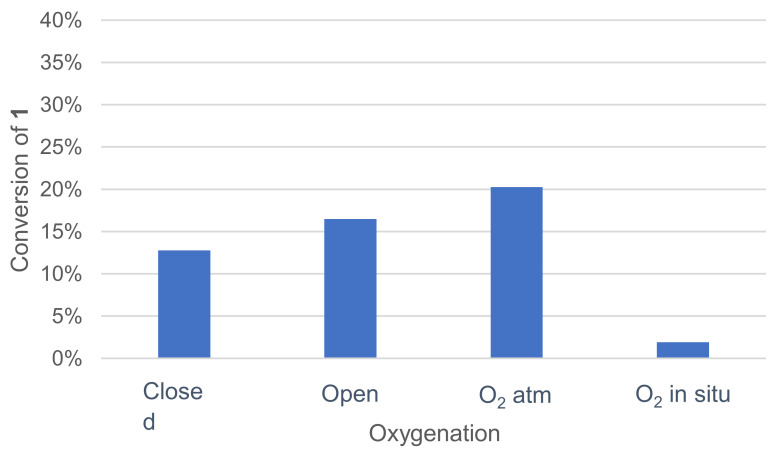
Effect of oxygenation on the conversion of **1** in the laccase-catalyzed oxidation (LA 5%*_w/w_*, HOBt 10 mol%, acetate buffer pH5 0.5M, DMF 5%*_v/v_*, incubation for 24 h, 30 °C, 200 rpm).

Various oxygenation methods were tested: closed vessel, open vessel at ambient atmosphere, vessel under an oxygen atmosphere and oxygenation by controlled in situ catalase degradation of hydrogen peroxide (Figure 5). Once again, the effect was quickly assessed by monitoring the remaining **1** in the crude reaction mixture after extraction and GC/MS analysis.

Oxygenation with the catalase–H_2_O_2_ system did not improve **1** conversion. A noticeable enhancement of the conversion was observed when the reaction was run under an oxygen atmosphere.

#### 3.1.5. Effect of Reaction Time

Increasing the reaction time usually allows a reactive system to reach the total conversion if there is no limitation by reagents quantities. The acylation of palmarosa essential oil by a lipase from *Candida rugosa* was reported to reach a 46% conversion after 24 h, whereas total conversion was obtained after 96 h. [44] The effect of reaction time was measured by a kinetic plot, and the conversion of **1** was assessed by monitoring the remaining **1** in the crude reaction mixture after extraction and GC/MS analysis.

**Figure 6:**
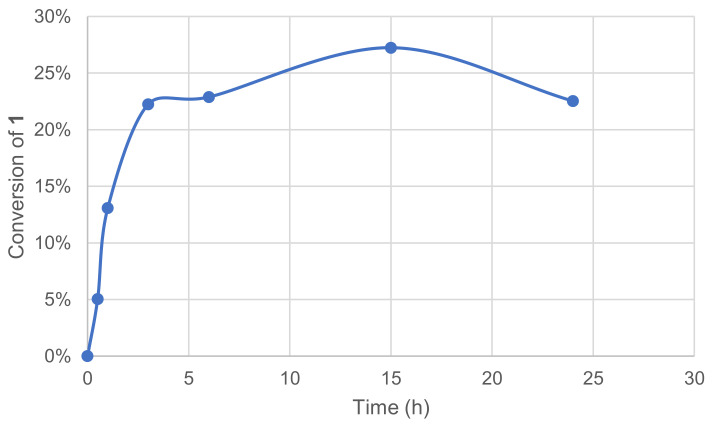
Effect of time reaction on the conversion of 1 in the laccase-catalyzed oxidation (LA 10%*_w/w_*, HOBt 20 mol%, acetate buffer pH5 0.25 M, O_2_ atmosphere, incubation 40 °C, 200 rpm).

The assays showed that the conversion raised significantly over a 5h-period with an initial reaction rate of ca. 32 mM·h^−1^. Conversion plateau was reached at 25–30% after 10 h, and this might be due to enzyme and/or mediator deactivation. The preincubation of some components was thus investigated mostly to evaluate a possible inhibiting effect of substrate **1** on the enzymatic system. The enzyme and **1** were thus engaged in our conditions in the absence of a mediator, while in parallel, the mediator and 1 were engaged similarly in the absence of the enzyme. After 24 h, the laccase–mediator system was completed by the addition of the missing component and the reaction was run for another 24 h. No significant differences were observed between these two experiments and the one in Figure 6.

#### 3.1.6. Effect of Enzyme Source and Concentration

Three laccases were used in this study to compare their activity. Laccase LTv was purchased from Sigma Aldrich (laccase from *Trametes versicolor*), laccase LA is a non-GMO enzyme purchased from Amano enzyme (laccase M120) and laccase LEH was obtained from ISM2 laboratory (Aix-Marseille Université) and produced by heterologous expression. These variable sources exhibited different specific activities that are not always precisely determined by vendors, which makes it difficult to design experiments for a comparison purpose. An SDS-PAGE test was performed on LA and LTv, showing that enzymes purity was rather low (see Supplementary Materials). In these reactivity tests, laccases were used in varying weight% relative to ME, and a comparison was made by measuring oxidation products in the crude reaction mixture after extraction and GC/MS analysis. The effect of substrate concentration was assayed on another side and was found negligible (see Supplementary Materials).

**Figure 7:**
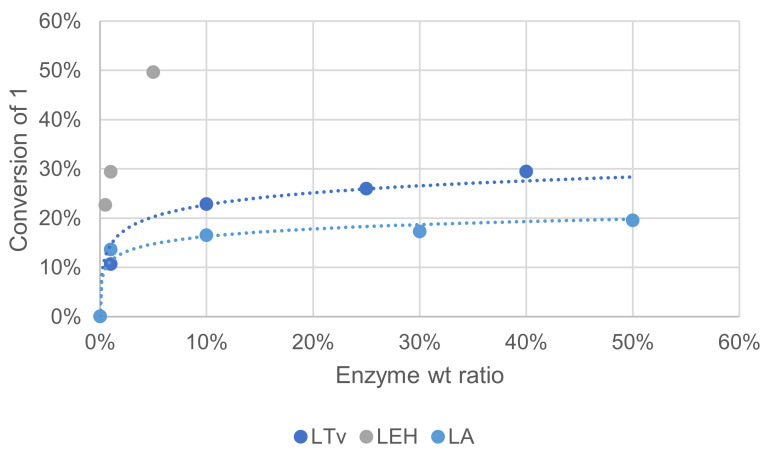
Effect of the enzyme source on the conversion of **1** in the laccase-catalyzed oxidation (laccase 1—50%*_w/w_*, HOBt 10 mol%, acetate buffer pH5 0.25 M, O_2_ atmosphere, incubation for 24 h, 40 °C, 200 rpm).

Increasing enzyme quantity could help reach a better conversion into oxidation products, but the increase was somehow limited beyond 0.25. Laccase LEH, shown to be of the highest purity, was clearly superior for this reaction with oxidation products formed with more than 50% yield with only 5 wt%. Mediator quantity is also an important parameter in relation to enzyme quantity. Compared to the conditions of Figure 7, when the reaction was performed with 4-fold HOBt as the mediator, conversion of **1** increased by ca. 2-fold (not shown).

#### 3.1.7. Effect of the Mediator Type

**Figure 8:**
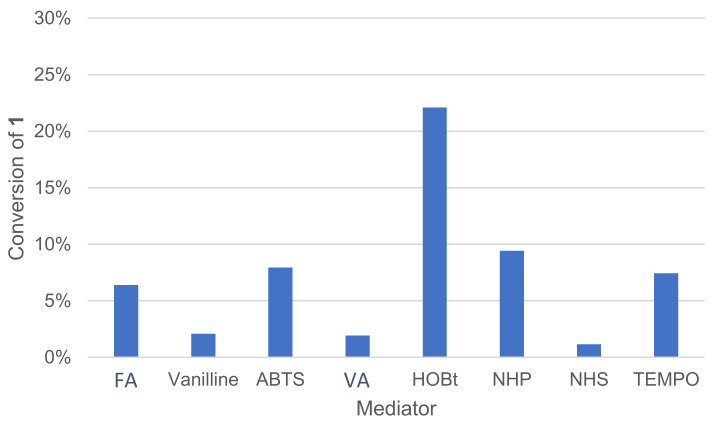
Effect of the mediator type on the conversion of **1** in the laccase-catalyzed oxidation (LA 10%*_w/w_*, mediator 20 mol%, acetate buffer pH5 0.25 M, O_2_ atmosphere, incubation for 24 h, 40 °C, 200 rpm). NPH: N-hydroxyphthalimide, NHS: N-hydroxysuccinimide, VA: violuric acid, FA: ferulic acid.

Various types of mediators are already described in the literature and typically used in combination with laccases, such as 1-hydroxybenzotriazole (HOBt), TEMPO and ABTS [18]. Their role is to link the enzyme with substrates that are not compatible for steric or electronic reasons. A mediator is a molecule of low molecular weight, which is oxidizable by laccase to a high potential intermediate, and whose reduced and oxidized forms must be stable [14]. The mediator must not deteriorate during oxidation cycles nor deactivate the enzyme. After being oxidized by laccase, the mediator oxidizes the substrate in a non-enzymatic process. The mediator returns to its initial reduced form and is ready to perform a new cycle. These mediators have redox potential compatible with the enzyme as well as a broader substrate scope. All of those mediators are of synthetic origin. Natural mediators can also be used, such as violuric acid (VA) and vanillin [28]. A pool of natural and non-natural mediators was thus tested (Figure 8).

**Figure 9:**
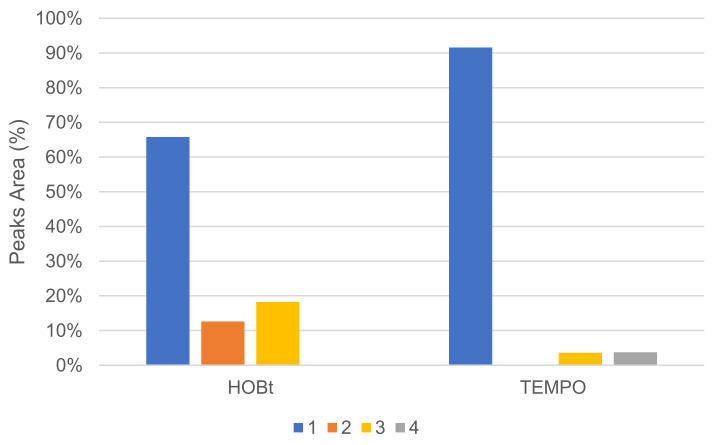
Comparison of oxidation products using HOBt or TEMPO as %area in the crude product determined by GC/MS (LA 10%*_w/w_*, mediator 50 mol%, acetate buffer pH5 0.25 M, O_2_ atmosphere, incubation for 24 h, 40 °C, 200 rpm).

The mediator type had an important effect on conversion, and no or very little reaction occurred in our conditions when using N-hydroxysuccinimide (NHS), vanillin, violuric acid (VA) and ferulic acid (FA). The profile of oxidation products distribution was also affected by mediator choice. In the same proportion, HOBt converted more **1** than TEMPO, but HOBt produced more oxidative cleavage products, such as veratraldehyde **2** and 3-(3,4-dimethoxyphenyl)prop-2-en-1-one **3** than TEMPO (Figure 9). TEMPO was also able to convert **1** into 1-(3,4-dimethoxyphenyl)prop-2-en-1-one **4**.

As organic molecules, mediators are extracted during work-up together with unconverted **1** and oxidation products. Using immobilized (supported) mediators could be an option as they can be easily separated from the reaction mixture by filtration. Supported HOBt and TEMPO were tested on **1** on our standard conditions but did not show any activity (not shown). In parallel, a positive control experiment was set using 4-methoxybenzyl alcohol oxidation [18]. Without a mediator, no conversion was observed. Free HOBt allowed for substrate oxidation as expected with 34% and the formation of resp. 18% and 12% oxidation products such as **3** and **4**. When using supported mediators, such as silica-bound TEMPO, PS-bound TEMPO and PS-bound HOBt, no reaction occurred, and the substrate was quantitatively recovered after work-up. Thus, we hypothesized that these supported mediators could not be activated by laccase within the binding pocket, eventually enabling substrate oxidation.

#### 3.1.8. Effect of Mediator Concentration

**Figure 10:**
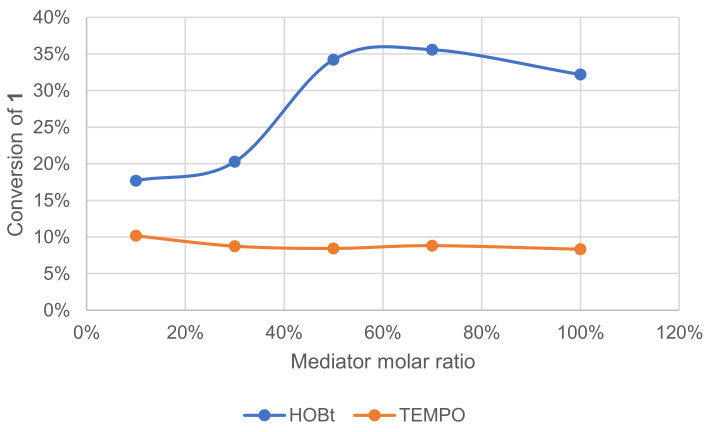
Effect of the mediator type and ratio on the conversion of **1** in the laccase-catalyzed oxidation (LA 10%*_w/w_*, mediator, acetate buffer pH5 0.25 M, O_2_ atmosphere, incubation for 24 h, 40 °C, 200 rpm).

The mediator in the laccase–mediator system is an intermediate that should switch between its oxidized and reduced form in a catalytic cycle. Following this scheme, each molecule of the mediator should be reusable endlessly. The effect of mediator concentration on the conversion of **1** was investigated (Figure 10). The conversion was monitored by GC/MS measurement of **1** after extraction of the crude reaction mixture. With HOBt, conversion increased with the ratio reaching a plateau at ca. 50 mol%. With TEMPO, however, conversion remained low regardless of its ratio.

#### 3.1.9. Hypothesis of a Fast Deactivation and Sequential Supply of Fresh Enzyme and Mediator

**Figure 11:**
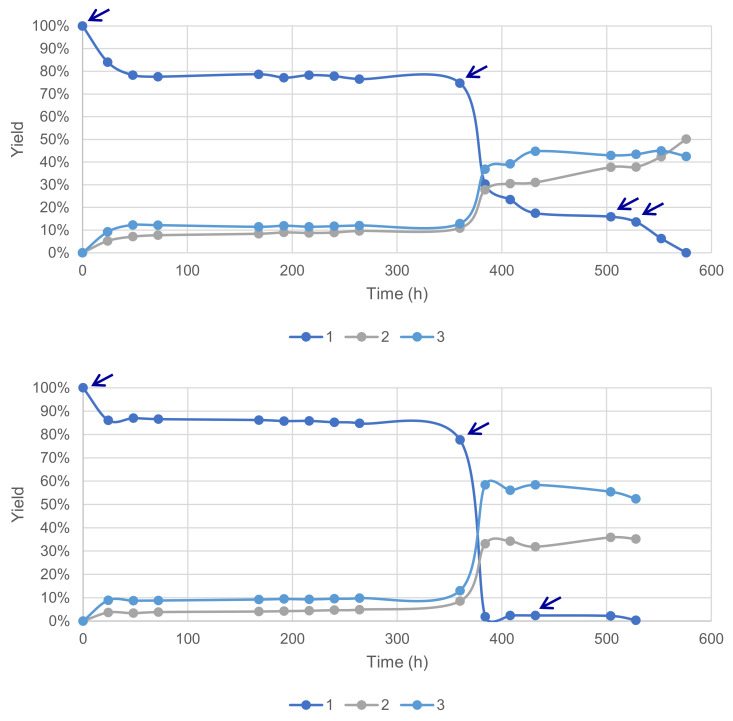
Sequential supply of fresh enzymes (**top**) and the mediator (**bottom**) marked with arrows for each addition of material (acetate buffer pH5, 6 mmol substrate **1** 0.12 M, O_2_ atmosphere, 40 °C, magnetic stirring).

Previous results obtained during this study suggested a fast deactivation of the laccase–mediator system in our conditions. This hypothesis was further tested with reactions conducted with a sequential supply of fresh enzymes and the mediator under time-course monitoring. A total of 4 additions of 5 wt% of laccase LA was necessary to reach 100% conversion and 93% yield of **2** and **3**, favorable to the former at 54% (Figure 11). Conversely, a total of 3 additions of 10 mol% HOBt allowed a 100% conversion and 87% yield, with a 60% selectivity in favor of **3**.

These results confirm the deactivation of both the enzyme LA, already described for a laccase from *Trametes villosa* with HOBt [24], but also the deactivation of HOBt, presumably by degradation.

### 3.2. Mechanistic Investigations

**Figure 12:**
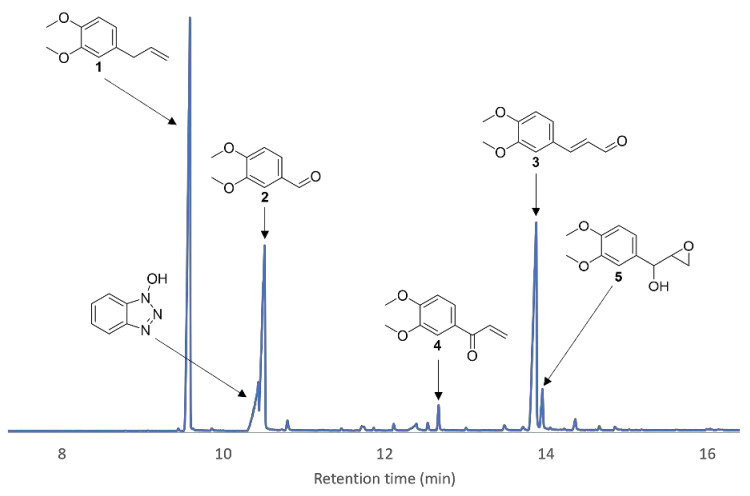
Zoom-in of a GC/MS chromatogram of the crude product after the enzymatic reaction of **1** with HOBt as a mediator. Five peaks were identified as oxidation products besides HOBt.

Identification of oxidation products of **1** was possible upon daily addition of enzyme and mediator until full conversion of **1** was attained (Figure 12). Isolation by chromatography on silica gel and confirmation by chemical synthesis allowed to identify compounds **2**, **3** and **4**.

**Figure 13:**
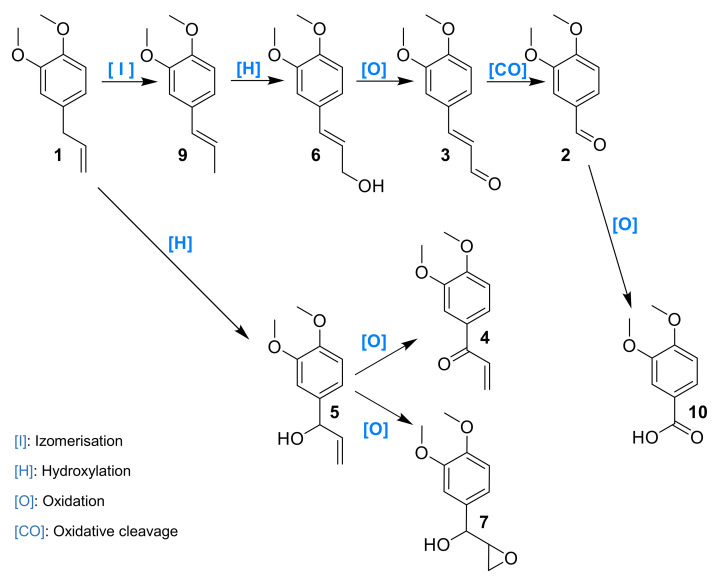
Hypothetical mechanism of the oxidation of **1** by the laccase–mediator system.

The reaction mechanism was hypothesized based on intermediates identified and their observed rate of formation/conversion (Figure 13). ME **1** could either oxidize on its allylic position to **5** or first isomerize into **9** and then further oxidize to **6**. A concerted isomerization/oxidation could also convert **1** into **6**. The oxidation of activated alcohol functions of **5** and **6** to their corresponding carbonyl compounds could thus take place leading to **4** and **3**, respectively. Veratraldehyde **2** could be either formed upon oxidative cleavage of all these intermediates and could eventually deliver benzoic acid derivative upon an additional oxidation step.

**Figure 14:**
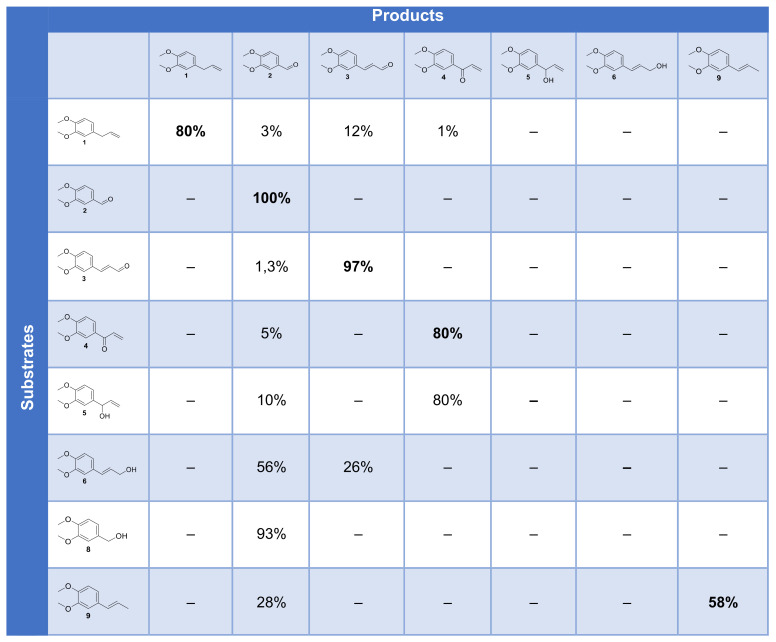
Cross-table of the conversion of substrates and intermediates by the laccase–mediator system.

Each step of this hypothetic mechanism was checked by the submission of the corresponding intermediate, either isolated, purchased or synthesized, to the enzymatic reaction, with HOBt as a mediator. Products and their proportion for each reaction are summarized in Figure 14.

ME **1** is mostly converted into **2**, **3** and **4**. Aldehyde **2** is not converted by the laccase–mediator system and recovered unchanged, which means that the benzoic derivative is not produced by our system. Compound **3** could not be oxidized by our system, ruling out that it could be an intermediate to **2**. Compound **4** remained mostly unconverted as well. Compound **5** could be oxidized to **4** but only to a limited extent oxidized to **2**, which also rules out this pathway. Compound **6** was predominantly oxidized to **2** upon oxidative cleavage and to 26% of 3. Compound **8** could be oxidized to **2** almost quantitatively, and compound **9** was partially oxidized to **2** by cleavage.

**Figure 15:**
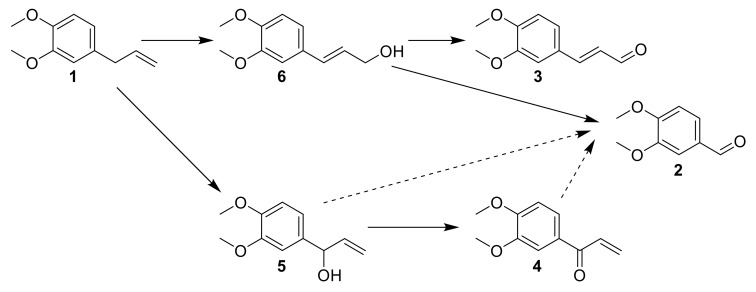
The proposed mechanism after data interpretation from intermediates reaction.

Considering the data obtained from intermediates reactivity, a proposed mechanism was elaborated (Figure 15). ME **1** is oxidized at the benzylic position to **5,** or both concomitantly isomerized and oxidized to **6**. Then, the oxidation of the alcohol function into aldehyde **3** is observed, or a direct oxidative cleavage to **2** happens. **5** could be oxidized to **4**, and these two last compounds could contribute to the formation of **2** by oxidative cleavage.

### 3.3. Scope and Limitations on Various Allylbenzene Derivatives

The reactivity of allylbenzene derivatives was investigated using commercially available substrates. Besides allylbenzene **11**, substituents on the benzenic ring exhibiting electron-withdrawing or electron-donating effects, such as 1-allyl-4-(dimethylamino)benzene **12**, 1-allyl-4-methoxybenzene **13**, 1-allyl-2-methoxybenzene **14**, 1-allyl-4-(trifluoromethyl)benzene **15**, eugenyl acetate **16**, allylpentafluorobenzene **17**, anethol **18**, isoeugenyl acetate **19** and dillapiole **20,** were also tested.

Selectivity in terms of oxidation products was examined for various electronic effects (see SI). No clear trend could be identified, and methyl eugenol **1** finally appeared as exhibiting the best compromise between the overall electron density and electronic effects.

## 4. Conclusions

The oxidation of allylbenzene derivatives by laccases and laccase–mediator systems was studied with a strong focus on methyl eugenol as a model substrate. The reaction was found to be quite slow, but total conversion could be obtained upon sequential addition of either laccase or a mediator. The oxidation proceeded by a concerted isomerization/hydroxylation of the propenyl side chain followed by two pathways, the former leading to cinnamaldehyde derivatives by further oxidation, and the latter to benzaldehyde derivatives by oxidative cleavage in a mild chemoenzymatic equivalent of ozonolysis. Alternatively, allylic hydroxylation could also be observed, marginally leading to cleavage products as well. The scope of the reaction was examined and was found to be dependent on substitution patterns and electronic effects; the best results being obtained with electron-rich methyl eugenol.

## Data Availability

Not available.

## Supplementary Materials

The following are available online. Detailed spectral data, procedures and NMR spectra.
