# Supplementary material for: Laccase-Catalyzed Oxidation of Allylbenzene Derivatives: Towards a Green Equivalent of Ozonolysis
Source: Molecules. 2021 Oct 6;26(19):6053. doi: 10.3390/molecules26196053 (PMC8512103; doi:10.3390/molecules26196053)
Supplement: Supplementary file 1 [file molecules-26-06053-s001.zip › molecules-1403768-SI.pdf]

molecules

*Supplementary Materials*

# Laccase-Catalyzed Oxidation of Allylbenzene Derivatives: Towards a Green Equivalent of Ozonolysis

**Mathilde Lecourt,[1] Giorgiana Chietera,[2] Bernard Blerot[2] and Sylvain Antoniotti[1]***

[1] Université Côte d'Azur, Institut de Chimie de Nice, CNRS, Parc Valrose, 06108 Nice cedex 2, France.
[2] LMR Naturals by IFF, Parc d'Activité les Bois de Grasse, 18 Avenue Joseph Honoré Isnard, 06130 Grasse, France.
* Correspondence: Sylvain Antoniotti (sylvain.antoniotti@univ-cotedazur.fr).

## 1/. Assessment of ME conversion by GC-MS

During the screening of the reaction conditions, the many experiments conducted required a fast method of assessment of the outcome of each individual reaction. We have chosen to use GC-MS to evaluate both the conversion of methyl eugenol **1** and the selectivity among the products formed. This method of analysis of the composition consisting in the integration of the chromatogram resulting from GC-MS analysis was validated by comparison with proton NMR. Mixtures of ME and known compounds in various proportions were prepared and analyzed by $^1$H NMR and GC-MS. The areas of the peaks of the chromatogram obtained by gas chromatography were compared with the proportions obtained by integration of the signals specific to each compound in the NMR spectrum. These two measurement methods were also compared with the molar quantities present in each sample (Figure S1).

**Copyright:** © 2021 by the authors. Submitted for possible open access publication under the terms and conditions of the Creative Commons Attribution (CC BY) license (https://creativecommons.org/licenses/by/4.0/).

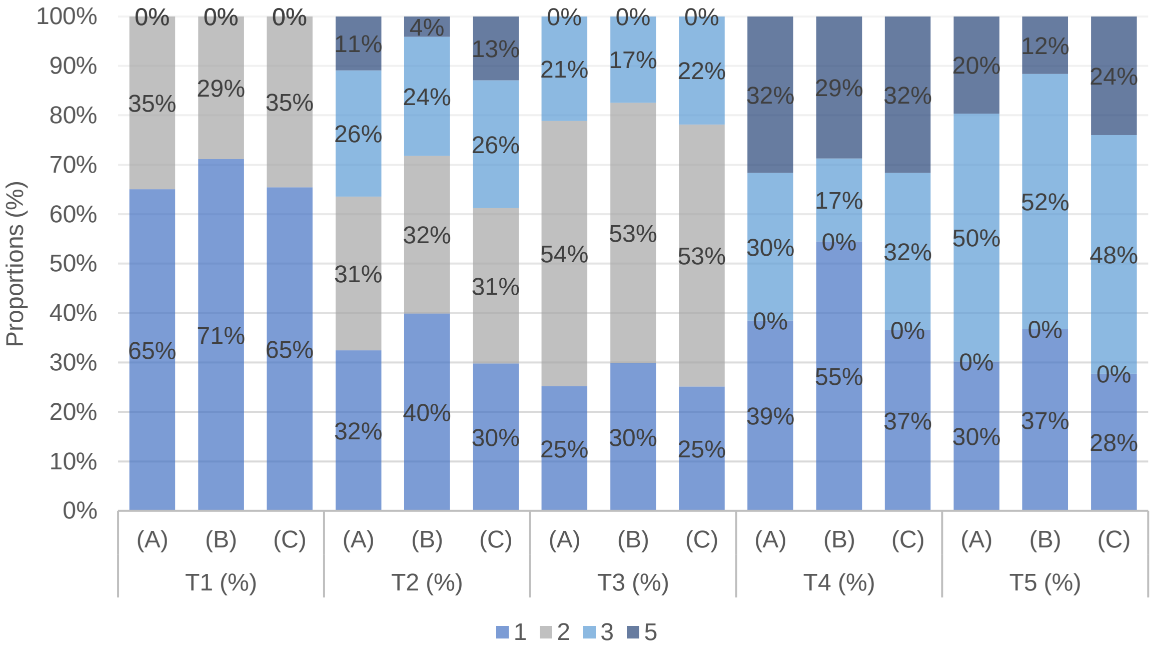


**Figure S1.** Comparison of proportions per sample in compounds 1, 2, 3, 5. (A): NMR, (B): GC-MS, (C): molar quantities, for samples T1 to T5.

The results validated our GC-MS approach as a first screen of the outcome of each oxidation reaction of **1** with relative error typically low.

## 2/. Effect of substrate concentration

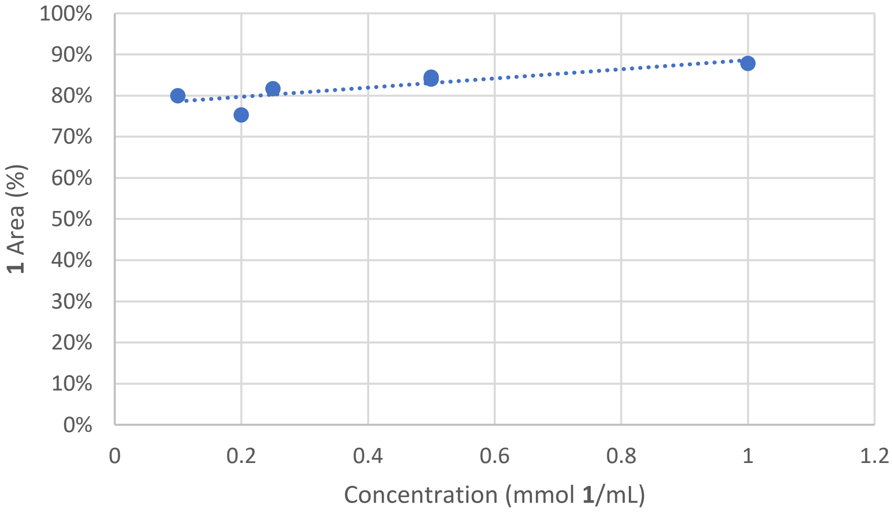


**Figure S2.** Effect of substrate concentration on conversion of 1 in the laccase-catalysed oxidation (LA 1%w/w, HOBt 10 mol%, acetate buffer pH5 4 mL, DMF 5%V/V, O2 atmosphere, incubation for 24 h, 30 °C, 200 rpm).

These results tend to show that the lower the concentration, the better the conversion, by a slight trend.

## 3/. Electrophoresis of enzymes used

Purity of enzymes used in this study were evaluated by SDS-PAGE technique. Catalase (Cat), laccases LA and LTv and peroxidase HRP (Figure S3).

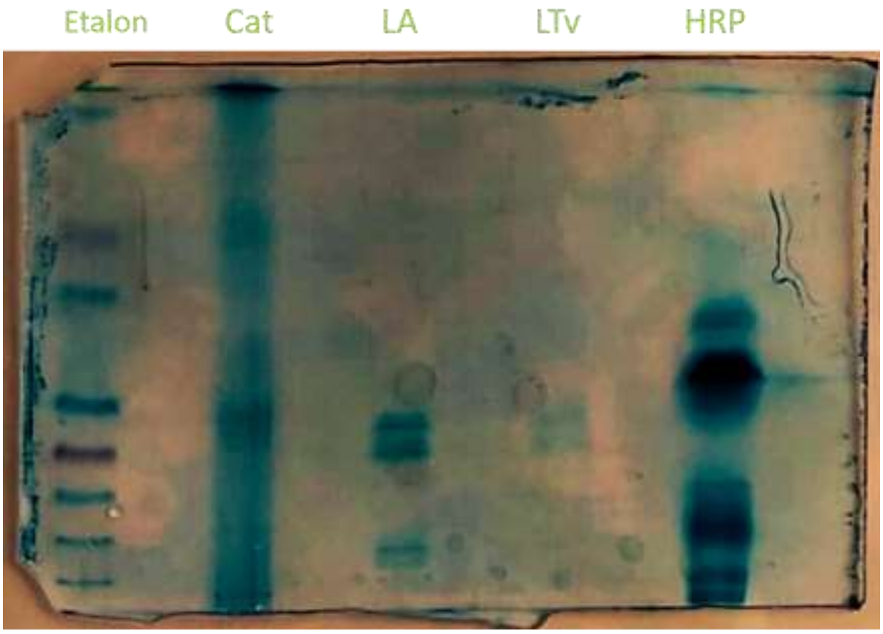


**Figure S3.** Gel electrophoresis by SDS-PAGE, denaturation at 90 °C and Coomassie blue dying (Quantities: Cat: 40 µg, LA: 133 µg, LTv: 100 µg, HRP: 20 µg).

## 4/. Electronic effects

**Table S1. Conversion and selectivity for substrates 11–20 compared with 1.**

| | Substrat M | Conversion | M+16 | M+14 | M-12 |
|---|---|---|---|---|---|
| Allyl substrates | 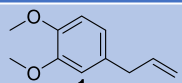 **1** | 33% | – | 18% | 4% |
| | 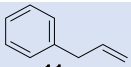 **11** | 100% | 24% | 68% | 5% |
| | 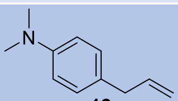 **12** | 4% | – | 2% | – |
| | 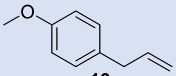 **13** | 5% | – | 2% | |
| | 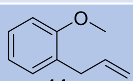 **14** | 10% | 7% | – | – |
| | 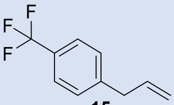 **15** | 15% | 4% | 6% | 1% |
| | 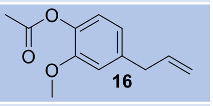 **16** | 5% | 2% | – | – |
| | 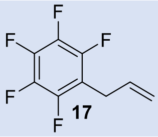 **17** | 10% | 7% | – | – |
| | 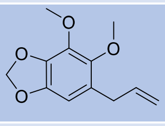 **20** | 88% | – | 5% | 7% |
| Propenyl substrates | 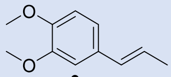 **9** | 25% | – | – | 11% |

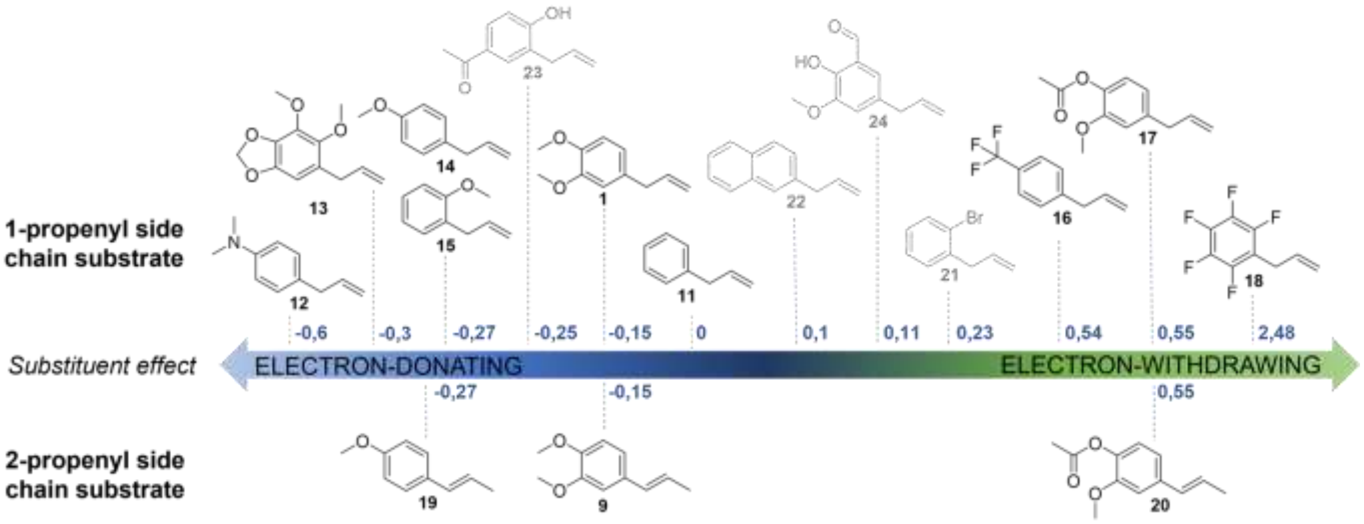


## 5/. Synthesis of substrates and intermediates

*1-(3,4-dimethoxyphenyl)prop-2-en-1-one* **4**

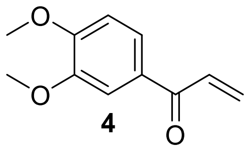

Name: 1-(3,4-dimethoxyphenyl)prop-2-en-1-one
CAS: 33731-40-7
Formula: $C_{11}H_{12}O_3$
MW (g/mol): 192,21
Aspect: Yellow solid

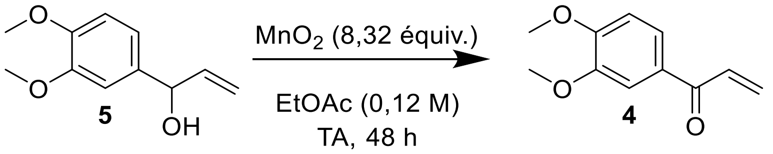


In a round-bottomed flask of 100 mL is introduced 1-(3,4-dimethoxyphenyl)prop-2-en-1-ol **5** (508 mg, 2.61 mmol), in EtOAc (22 mL) and manganese oxide (0.94 g, 4.16 equiv.). The reaction mixture is then mechanically stirred at room temperature and the reaction progress is monitored by TLC. Completion was achieved in 48 h after a second addition of $MnO_2$ (0.94 g, 4.16 equiv.). After filtration over Celite®545 and then silica gel, the filtrate is dried over magnesium sulfate, evaporated and residue purified by column chromatography.

Yield after purification: 44%

References[1–3]

$^1$H NMR (400 MHz, $CDCl_3$) δ (ppm) 7.61 – 7.56 (m, 2H, $H_{ortho}$), 7.19 (dd, *J* = 17.0, 10.5 Hz, 1H, $H_1$), 6.90 (d, *J* = 8.4 Hz, 1H, $H_{méta}$), 6.43 (dd, *J* = 17.0, 1.8 Hz, 1H, $H_3$), 5.87 (dd, *J* = 10.5, 1.8 Hz, 1H, $H_3$), 3.96 & 3.95 (s, 3H, $OCH_3$).

$^{13}$C NMR (100 MHz, $CDCl_3$) δ (ppm) 189.28, (C=O), 153.57 & 149.40 (CAr-$OCH_3$), 132.07, (CH=), 130.56, (CAr), 129.39, ($CH_2$), 123.56, (CHAr), 110.92, (CHAr), 110.11, (CHAr), 56.23, ($OCH_3$), 56.15, ($OCH_3$).

MS m/z (%): 192 ($M^{+\bullet}$, 50), 165 (100), 122 (20), 107 (22), 91 (20), 79 (78), 77 (40), 63 (25), 55 (60), 51 (39).

$^{1}$H NMR 1-(3,4-dimethoxyphenyl)prop-2-en-1-one **4**

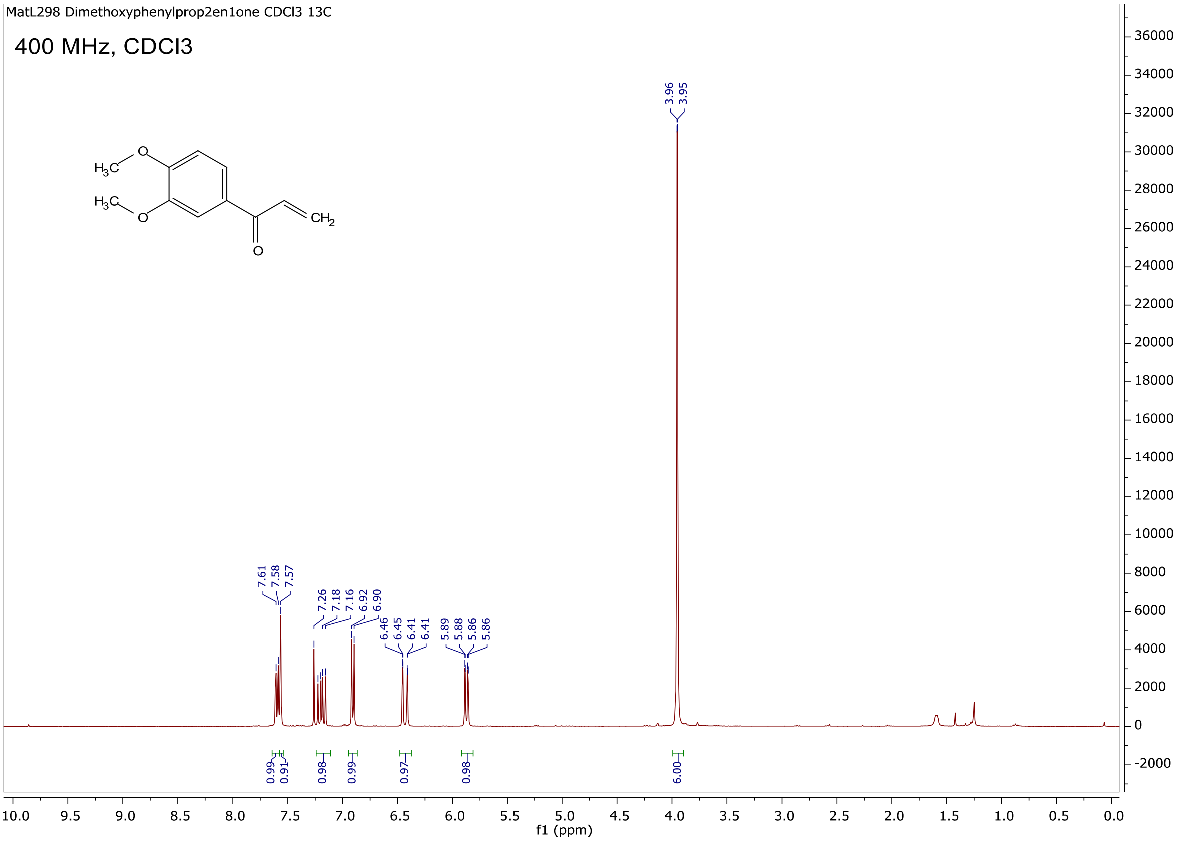


$^{13}$C NMR 1-(3,4-dimethoxyphenyl)prop-2-en-1-one **4**

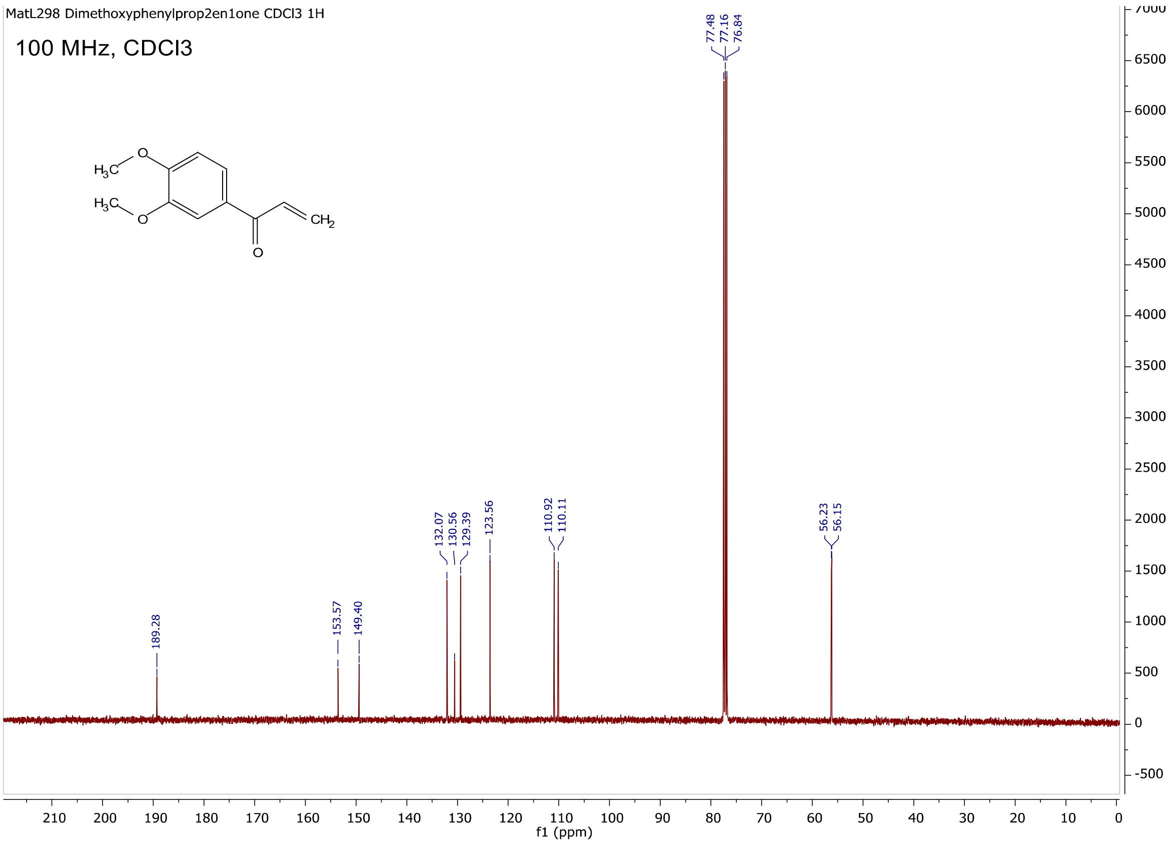

*Synthesis of 1-(3,4-dimethoxyphenyl)prop-2-en-1-ol* **5**

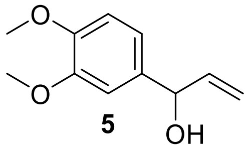


Name: 1-(3,4-dimethoxyphenyl)prop-2-en-1-ol
CAS: 31706-95-3
Formula: $C_{11}H_{14}O_3$
MW (g/mol): 194,23
Aspect: yellow liquid

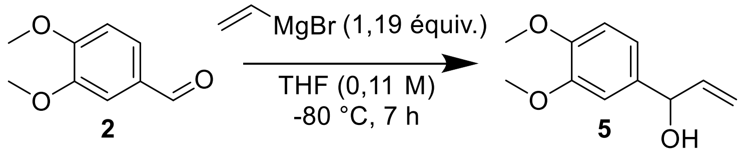


In a round-bottomed flask of 100 mL is introduced veratraldehyde **2** (1.43 g, 8.66 mmol) under a nitrogen atmosphere. Freshly distilled THF is added (80 mL) and the mixture cooled at -80 °C. Vinyl magnesium bromide solution in hexanes is then slowly added (10.3 mL, 10.3 mmol, 1,19 equiv.). The reaction mixture is then mechanically stirred until the return to room temperature and the reaction progress is monitored by TLC. Completion was achieved in 7 h and the reaction mixture hydrolyzed with a saturated aqueous $NH_4Cl$ solution, and further extracted with AcOEt (10 mL). After washing with a saturated aqueous NaCl solution, the organic layer is separated, dried over magnesium sulfate, evaporated and residue purified by column chromatography.

Yield after purification: 81%

References[4–7]

$^1$H NMR (400 MHz, $CDCl_3$) δ (ppm) 6.89 – 6.85 (m, 2H, $H_{ortho}$), 6.81 (m, 1H, $H_{méta}$), 6.02 (ddd, *J* = 17.2, 10.3, 5.9 Hz, 1H, $H_2$), 5.33 – 5.15 (m, 2H, $H_3$), 5.11 (dd, *J* = 5.9, 1.5 Hz, 1H, $H_1$), 3.85 & 3.84 (s, 3H, $OCH_3$).

$^{13}$C NMR (100 MHz, $CDCl_3$) δ (ppm) 149.21 & 148.71 (CAr-$OCH_3$), 140.37, (CH=), 135.41, (CAr), 118.76, (CHAr), 115.01, ($CH_2$), 111.13, (CHAr), 109.59, (CHAr), 75.18, (CH-OH), 56.04, ($OCH_3$), 55.95, ($OCH_3$).

MS m/z (%): 194 ($M^{+•}$, 97), 165 (29), 163 (62), 151 (33), 139 (100), 138 (27), 91 (34), 79 (31), 77 (36), 55 (51)

$^{1}$H NMR 1-(3,4-dimethoxyphenyl)prop-2-en-1-ol **5**

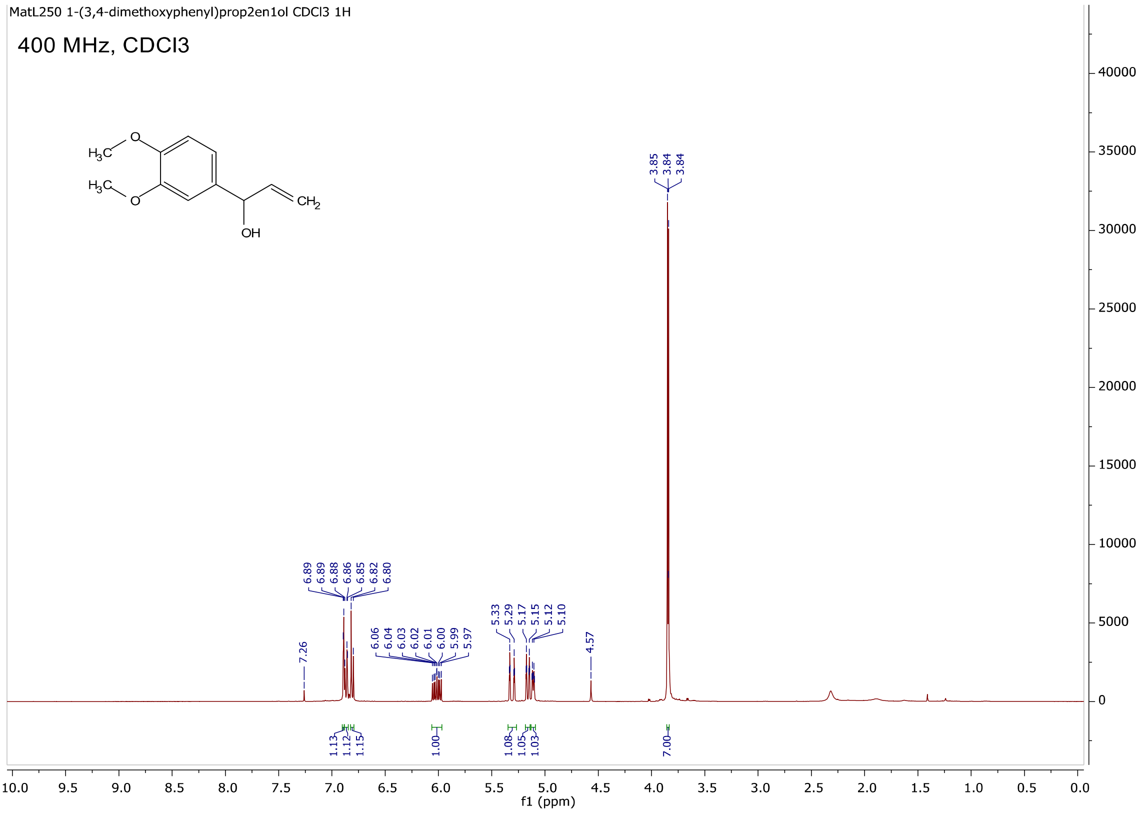


$^{13}$C NMR 1-(3,4-dimethoxyphenyl)prop-2-en-1-ol **5**

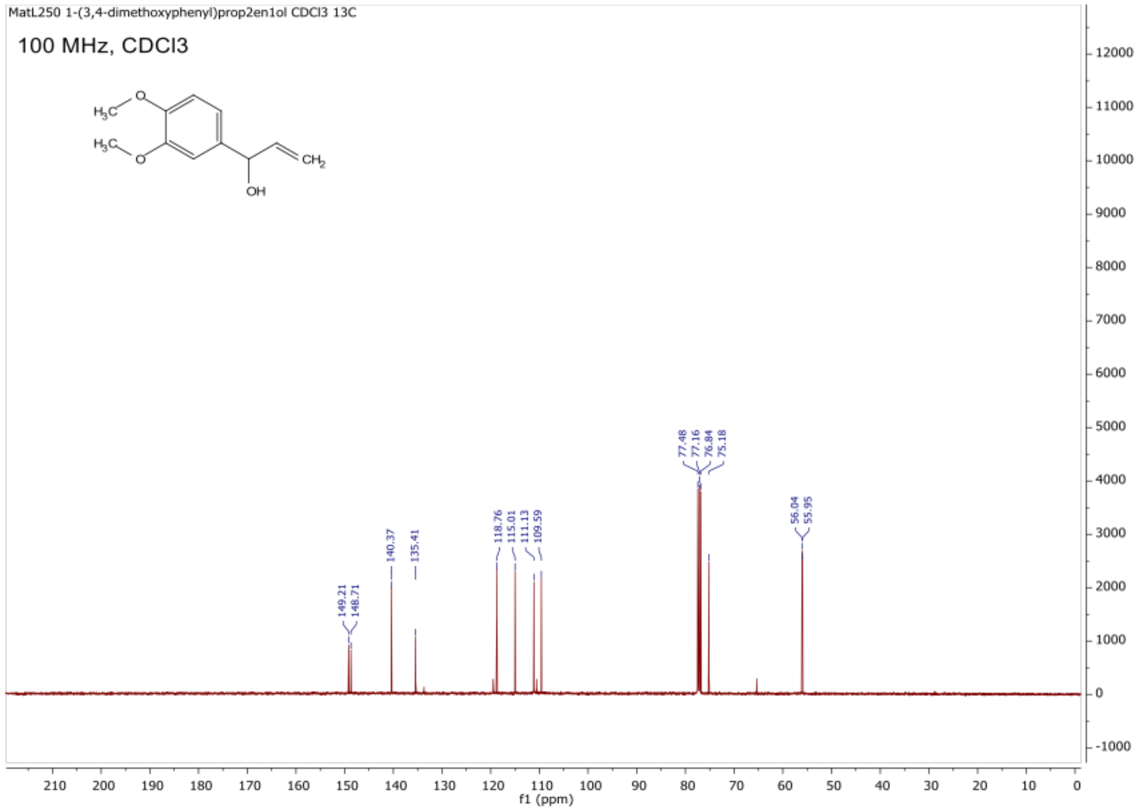

*Synthesis of 3-(3,4-dimethoxyphenyl)prop-2-en-1-ol* ***6***

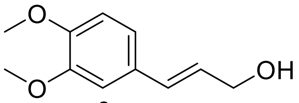


**6**

Name: 3-(3,4-dimethoxyphenyl)prop-2-en-1-ol
CAS: 18523-76-7
Formula: $C_{11}H_{14}O_3$
MW (g/mol): 194,23
Aspect: yellow solid

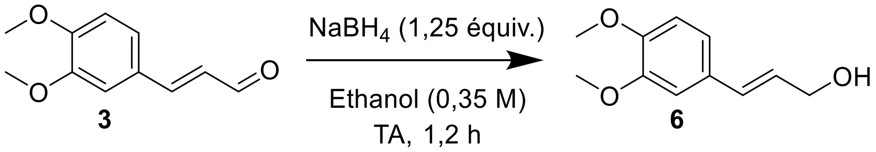


In a round-bottomed flask of 10 mL is introduced 3-(3,4-dimethoxyphenyl)prop-2-enal **3** (203 mg, 1.06 mmol,) in EtOH (3 mL). The reactor is placed under a nitrogen atmosphere and NaBH′ (50 mg, 1.32 mmol) is then slowly added. The reaction mixture is then mechanically stirred at room temperature and the reaction progress is monitored by TLC. Completion was achieved in 1.2 h and the solvent evaporated. The residue was dissolved in water (10 mL) and further extracted with AcOEt (3 x 15 mL). The organic layer is separated, dried over magnesium sulfate, evaporated and the product used without the need for further purification.

Yield: 100%

References[8,9]

$^1$H NMR (400 MHz, $CDCl_3$): δ (ppm) 6.98 – 6.88 (m, 2H, $H_{ortho}$), 6.82 (d, *J*=8.2 Hz, 1H, $H_{méta}$), 6.55 (d, *J*=15.9 Hz, 1H, $H_3$), 6.25 (dt, *J*=15.9, 5.8 Hz, 1H, $H_2$), 4.31 (d, *J*=5.8, 2H, $H_1$), 3.90 & 3.88 (s, 3H, $OCH_3$).

$^{13}$C NMR (100 MHz, $CDCl_3$): δ (ppm) 149.15 & 149.04 (CAr-$OCH_3$), 131.27 (CAr), 129.86 (CH=), 126.67 (=CH), 119.81 (CHAr), 111.24 (CHAr), 108.97 (CHAr), 63.95 ($CH_2$), 56.04, ($OCH_3$), 55.94 ($OCH_3$).

MS m/z (%): 194 ($M^{+\bullet}$, 68), 165 (14), 151 (100), 138 (53), 119 (16), 107 (13), 91 (33), 79 (14), 77 (26), 55 (13).

$^{1}$H NMR 3-(3,4-dimethoxyphenyl)prop-2-en-1-ol **6**

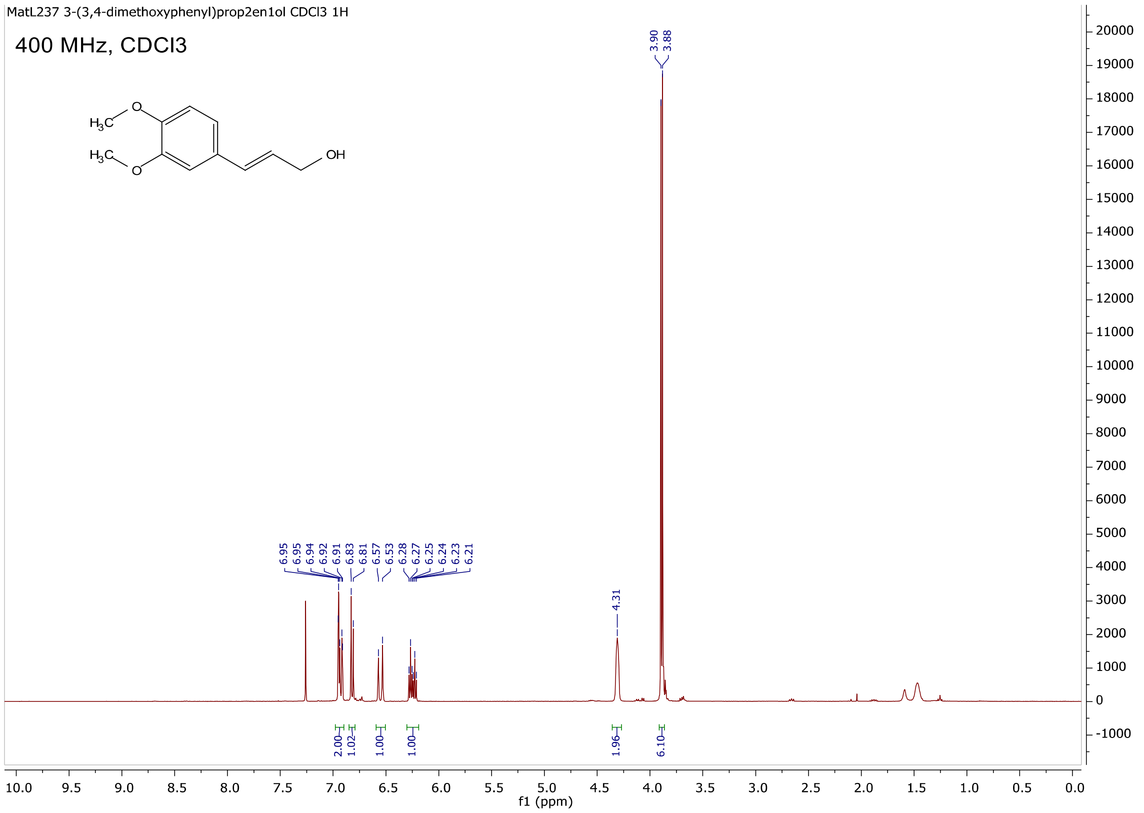


$^{13}$C NMR 3-(3,4-dimethoxyphenyl)prop-2-en-1-ol **6**

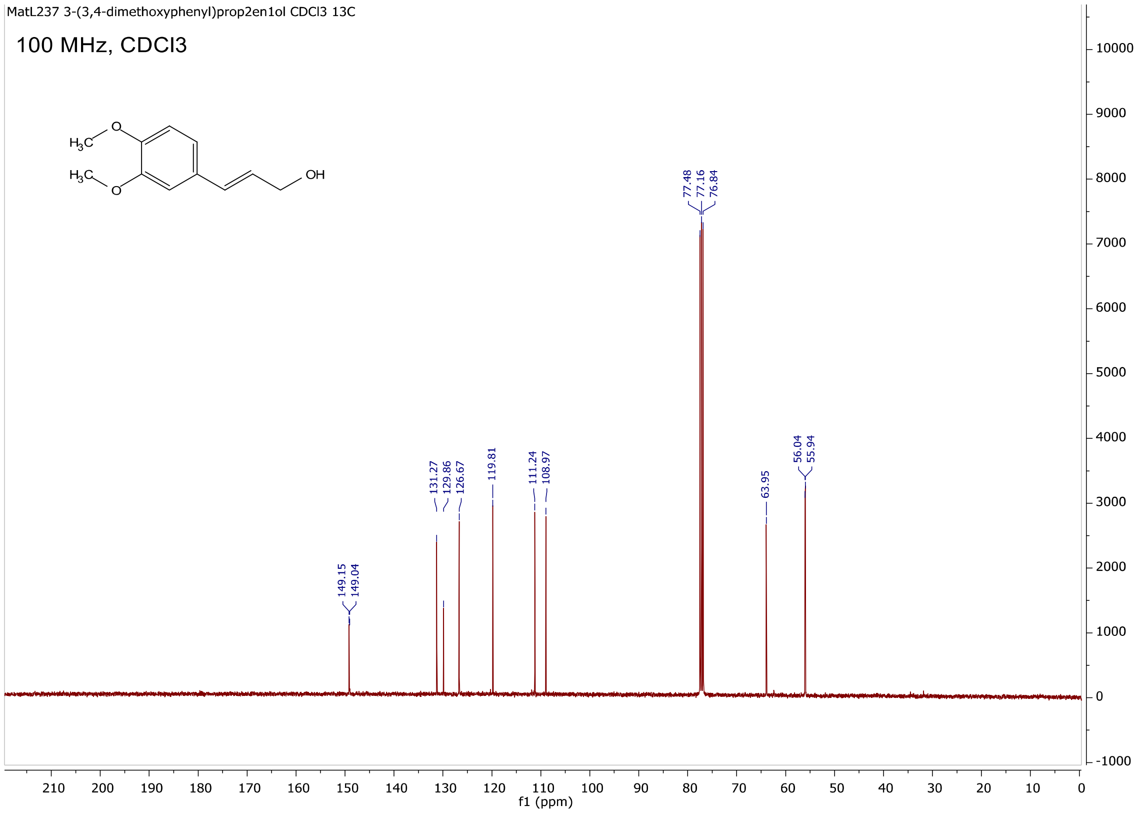

*1-(3,4-dimethoxyphenyl)-2,3-epoxypropan-1-ol* **5**

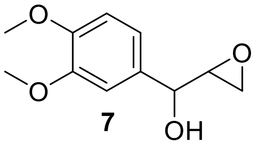


Name: 2,3-epoxy-1-(3,4-dimethoxyphenyl)propane-1-ol
CAS: none
Formula: $C_{11}H_{14}O_4$
MW (g/mol): 210.23

Isolated from reaction mixtures as a mixture of two diastereomers.

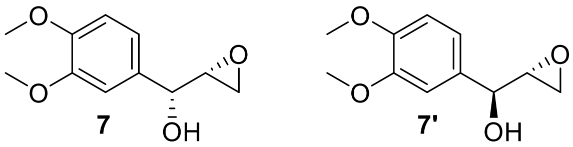


$^1$H NMR (400 MHz, $CDCl_3$) δ (ppm) 6.99 – 6.85 (m, 3H, $H_{Ar}$), 4.87 & 4.44 (d, 1H, $H_1$ (*cis*:*trans* 30:70)), 3.91 & 3.81 (s, 3H, $OCH_3$), 3.23 (m, 1H, $H_2$), 2.77 – 2.97 (m, 2H, $H_3$).
$^{13}$C NMR (100 MHz, $CDCl_3$) δ (ppm) 149.36 & 149.12 (CAr-$OCH_3$), 149.31 & 149.16 (C′Ar-$OCH_3$), 132.99, (CAr), 132.09, (C′Ar), 118.92, (C′HAr), 118.81, (CHAr), 111.25, (CHAr), 109.68, (C′HAr), 109.58, (CHAr), 109.55, (C′HAr), 74.27, (C-OH), 70.84, (C′-OH), 56.09 – 56.05 & 56.02, (CH, $OCH_3$, $OCH_3$), 56.09 – 56.05 & 55.17, (C′H, OC′$H_3$, OC′$H_3$), 45.50, ($CH_2$); 43.79, (C′$H_2$).
MS m/z (%): 210 ($M^{+\bullet}$,35), 167 (100), 165 (14), 151 (54), 139 (67), 124 (23), 108 (19), 107 (15), 77 (25), 65 (15).

References[10,11]

$^{1}$H NMR 1-(3,4-dimethoxyphényl)-2,3-epoxypropan-1-ol **5**

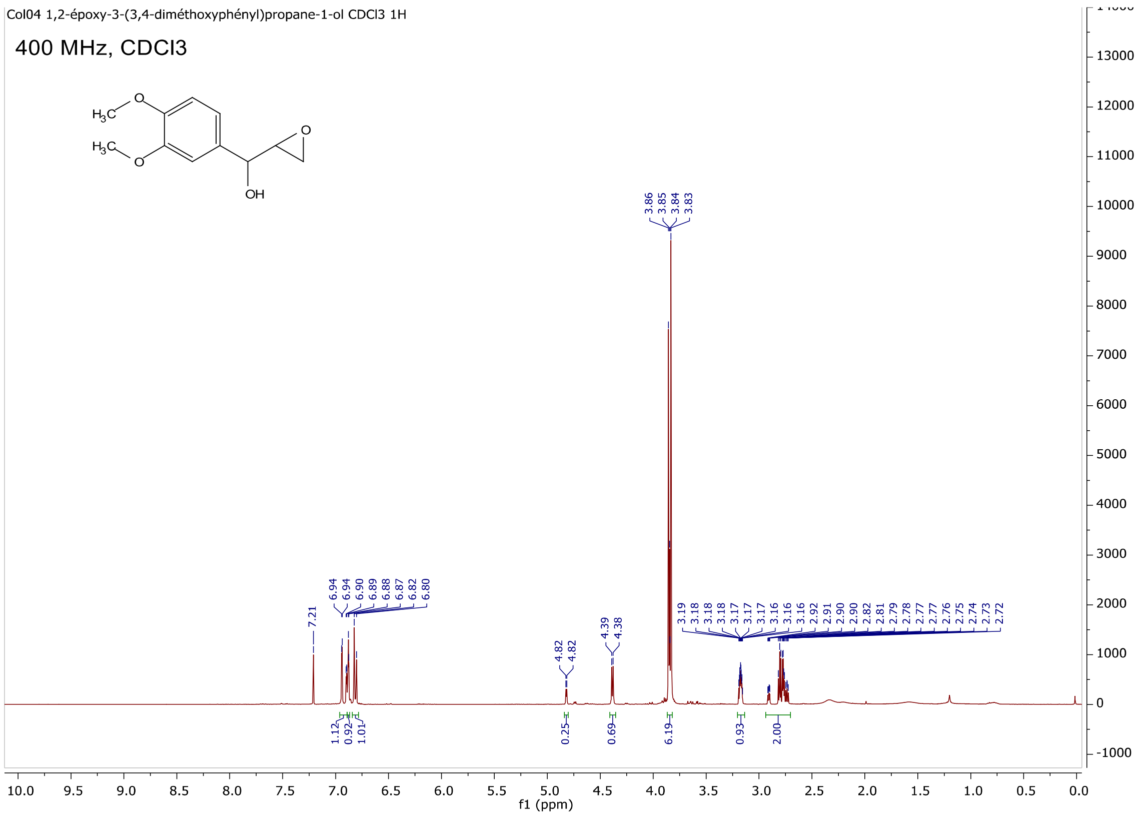


$^{13}$C NMR 1-(3,4-dimethoxyphenyl)-2,3-epoxypropan-1-ol **5**

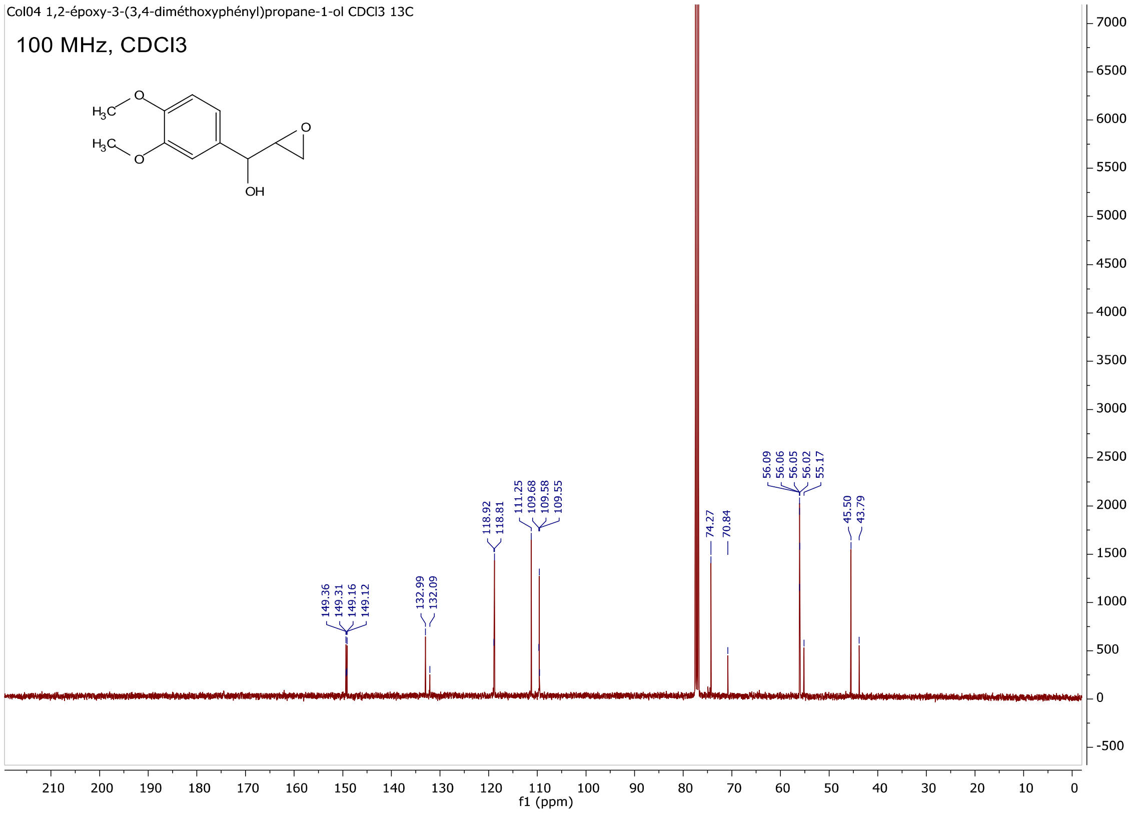

Dept 135 1-(3,4-dimethoxyphenyl)-2,3-epoxypropan-1-ol **5**

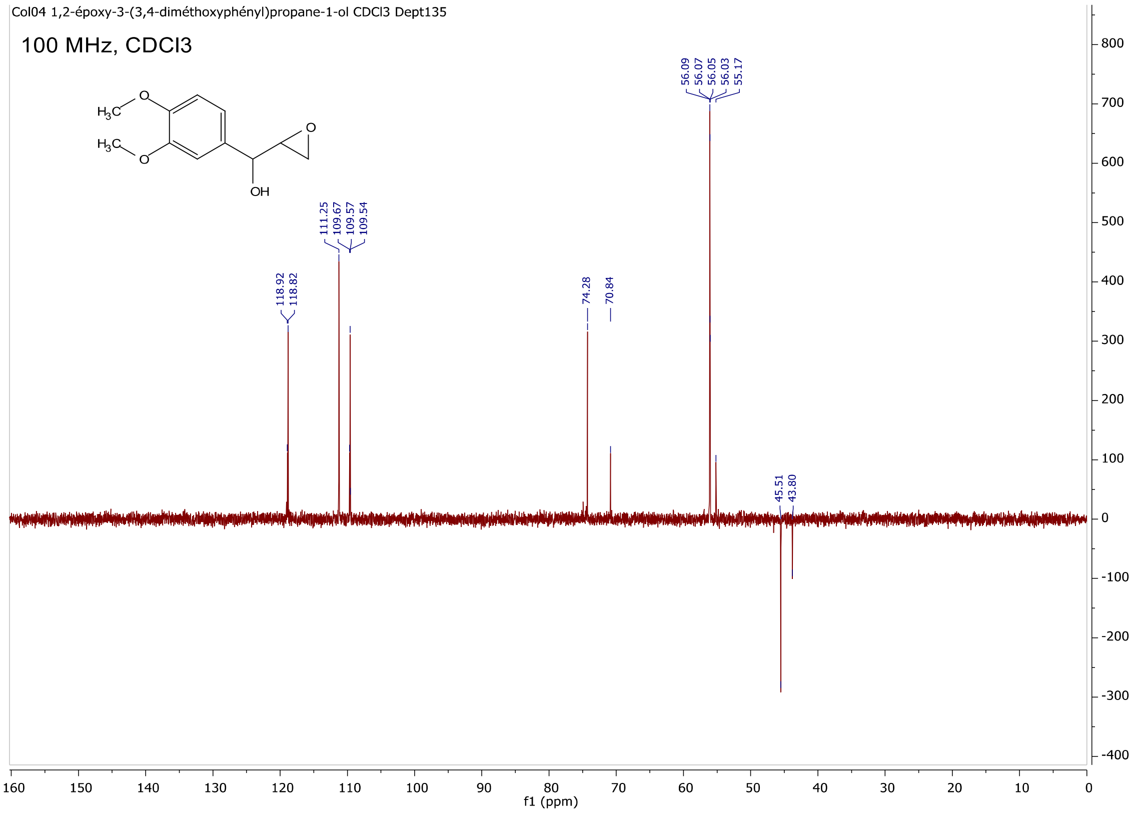


COSY 1-(3,4-dimethoxyphenyl)-2,3-epoxypropan-1-ol **5**

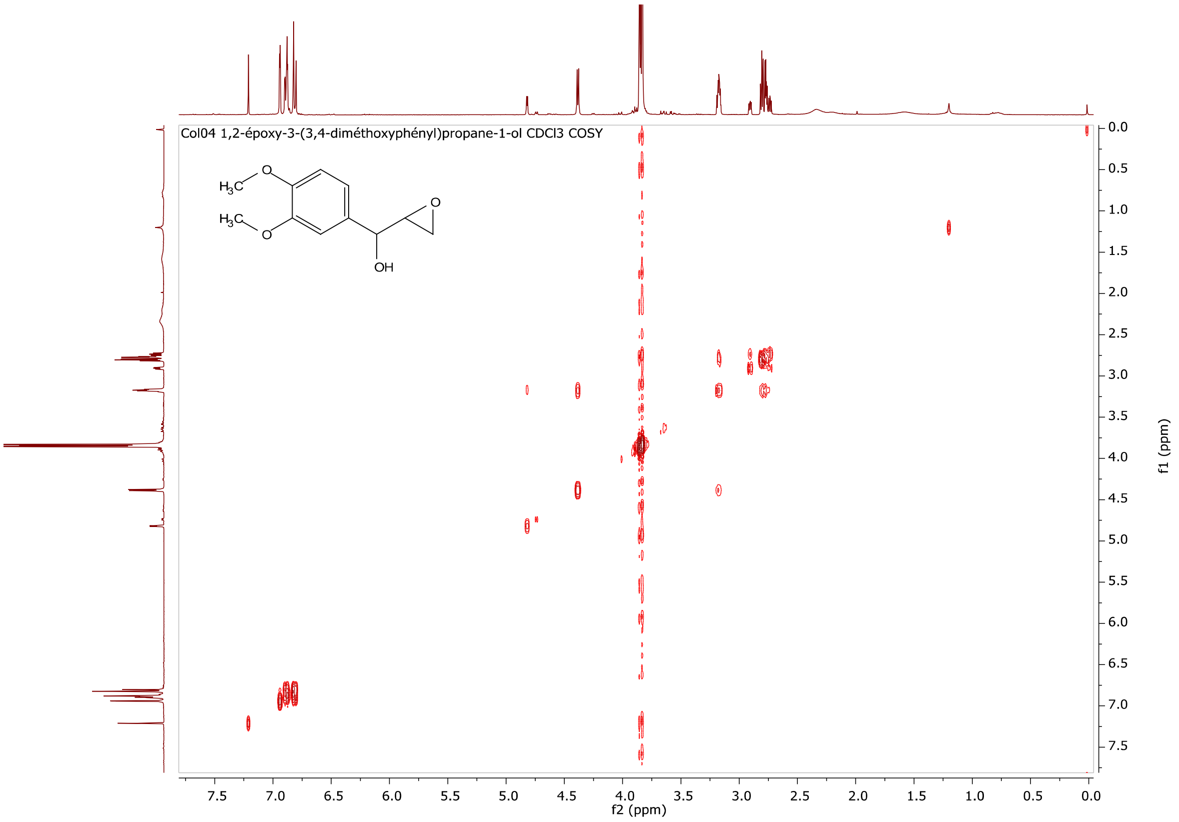

HSQC 1-(3,4-dimethoxyphenyl)-2,3-epoxypropan-1-ol **5**

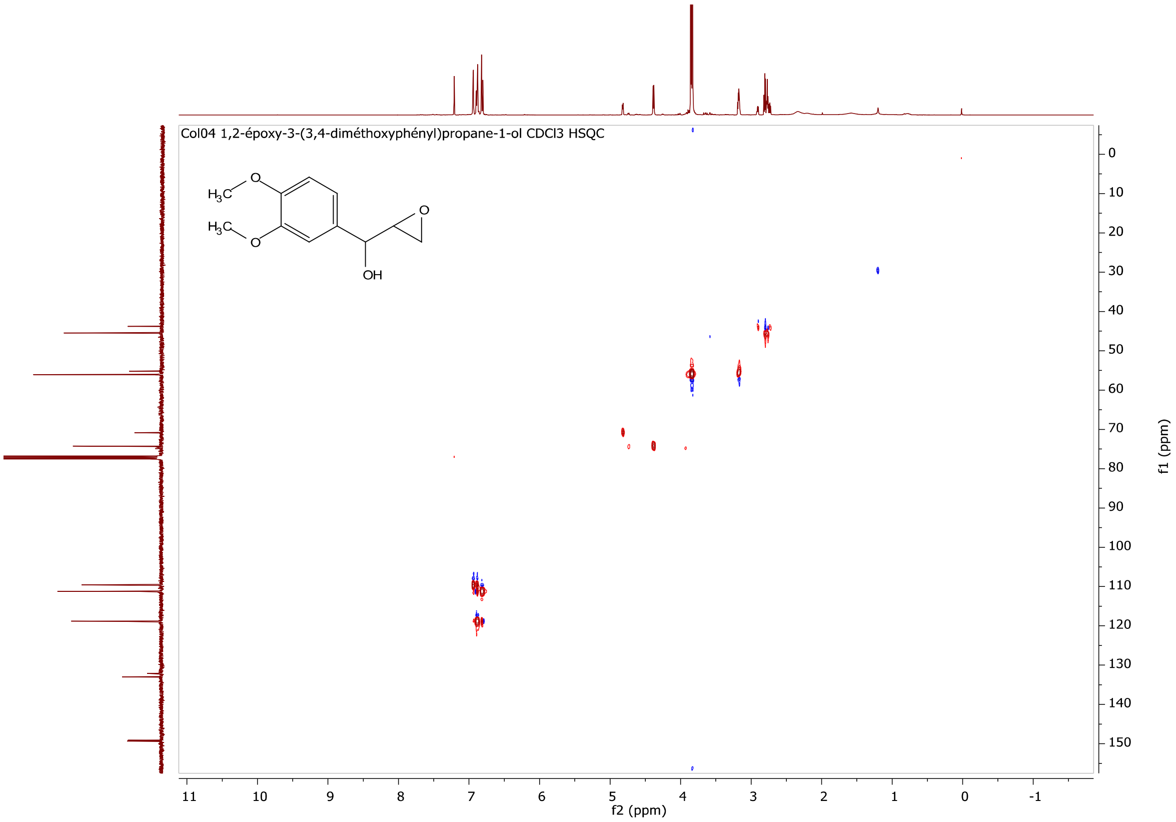


HMBC 1-(3,4-dimethoxyphenyl)-2,3-epoxypropan-1-ol **5**

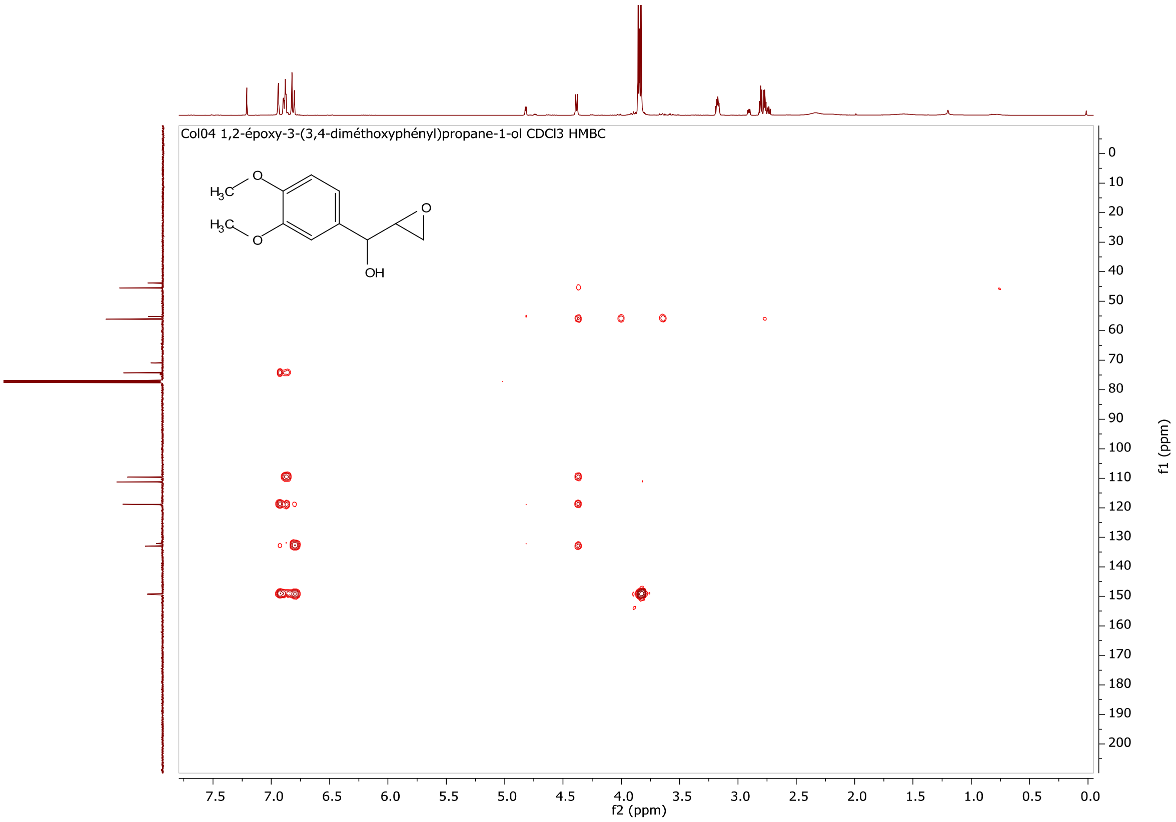

NOESY 1-(3,4-dimethoxyphenyl)-2,3-epoxypropan-1-ol **5**

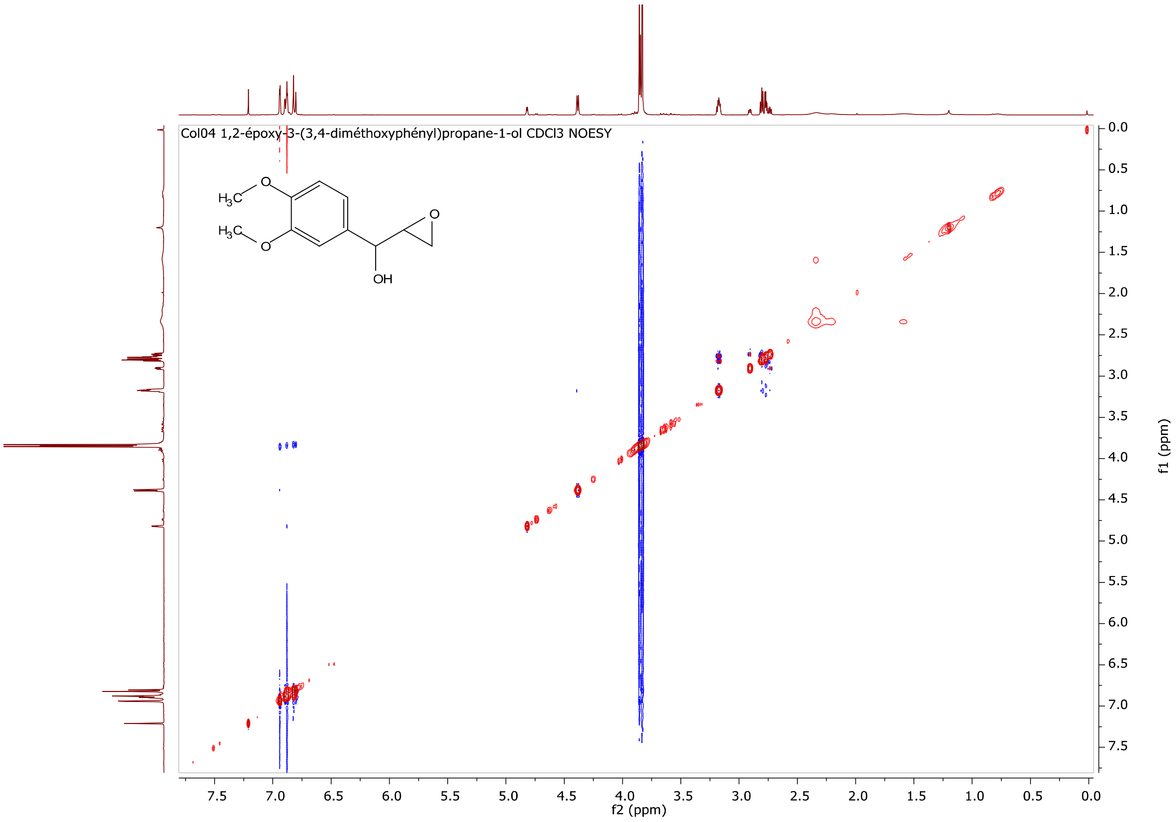

*Synthesis of 3,4-dimethoxybenzyl alcohol 8*

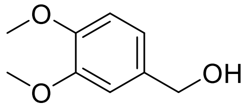


**8**

Name: 3,4-dimethoxyphenylmethanol
CAS: 93-03-8
Formula: $C_9H_{12}O_3$
MW (g/mol): 168.19
Aspect: colorless liquid

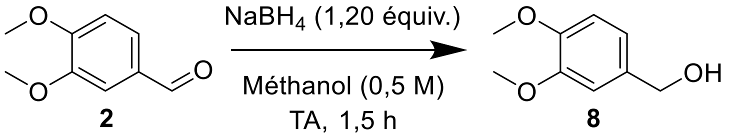


In a round-bottomed flask of 100 mL is introduced veratraldehyde **2** (1.68 g, 10.12 mmol) in MeOH (20 mL) and placed under a nitrogen atmosphere. $NaBH_4$ (459 mg, 12.13 mmol, 1.20 equiv.) is then added and the reaction mixture mechanically stirred at room temperature. Completion was achieved in 1.5 h as indicated by TLC. After evaporation of MeOH, the residue was suspended in water and extracted with EtOAc (3x15 mL). The combined organic layers were dried over magnesium sulfate, evaporated and the product used without further purification.

Yield: 88%

References[12–17]

$^1$H NMR (400 MHz, $CDCl_3$) δ (ppm) 6.92 – 6.83 (m, 3H, $H_{Ar}$), 4.62 (s, 2H, $H_2$), 3.89 & 3.87 (s, 3H, $OCH_3$).

$^{13}$C NMR (100 MHz, $CDCl_3$) δ (ppm) 149.21 & 148.68 (CAr-$OCH_3$), 133.70, (CAr), 119.49, (CHAr), 111.16, (CHAr), 110.57, (CHAr), 65.39, ($CH_2$), 56.05, ($OCH_3$), 55.94, ($OCH_3$).

MS m/z (%) : 168 ($M^{+\bullet}$, 100), 151 (29), 139 (38), 137 (27), 109 (21), 97 (20), 93 (15), 79 (15), 77 (20), 65 (24).

$^1$H NMR 3,4-dimethoxybenzyl alcohol **8**

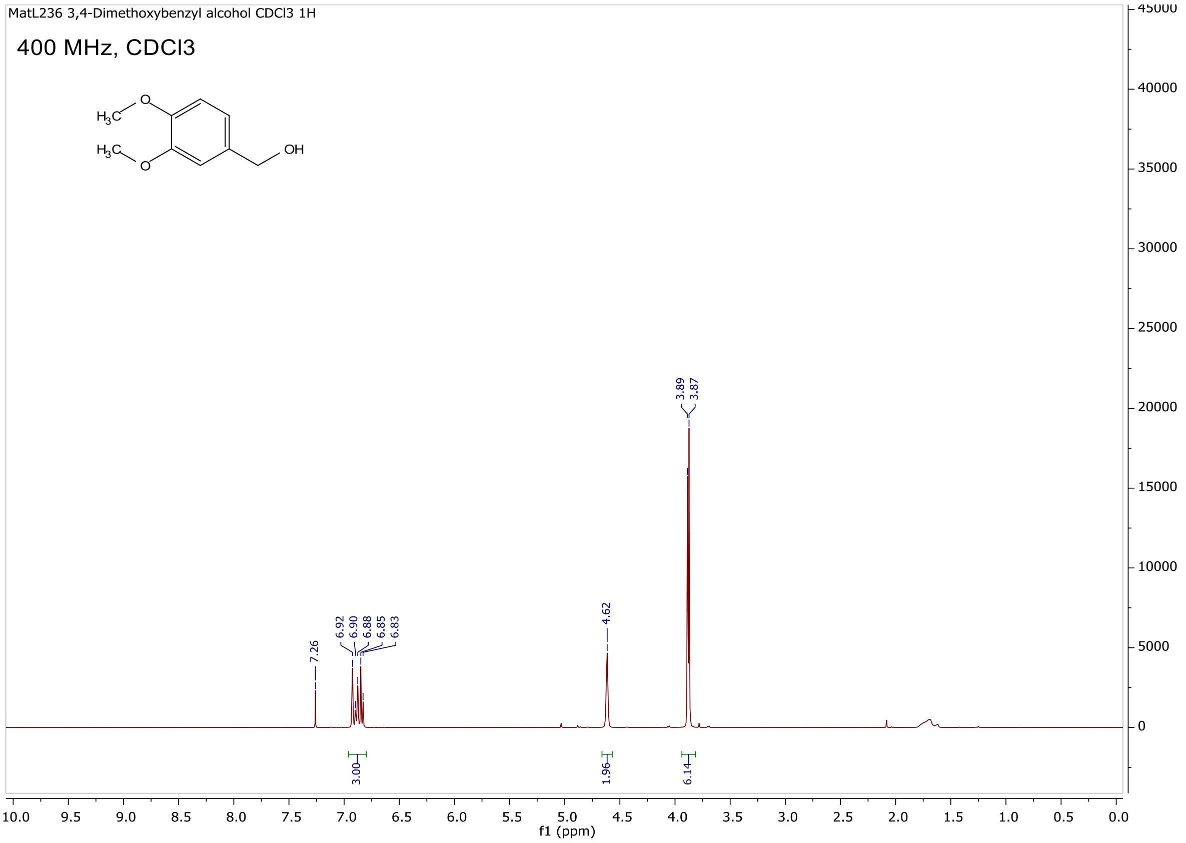

$^{13}$C NMR 3,4-dimethoxybenzyl alcohol **8**

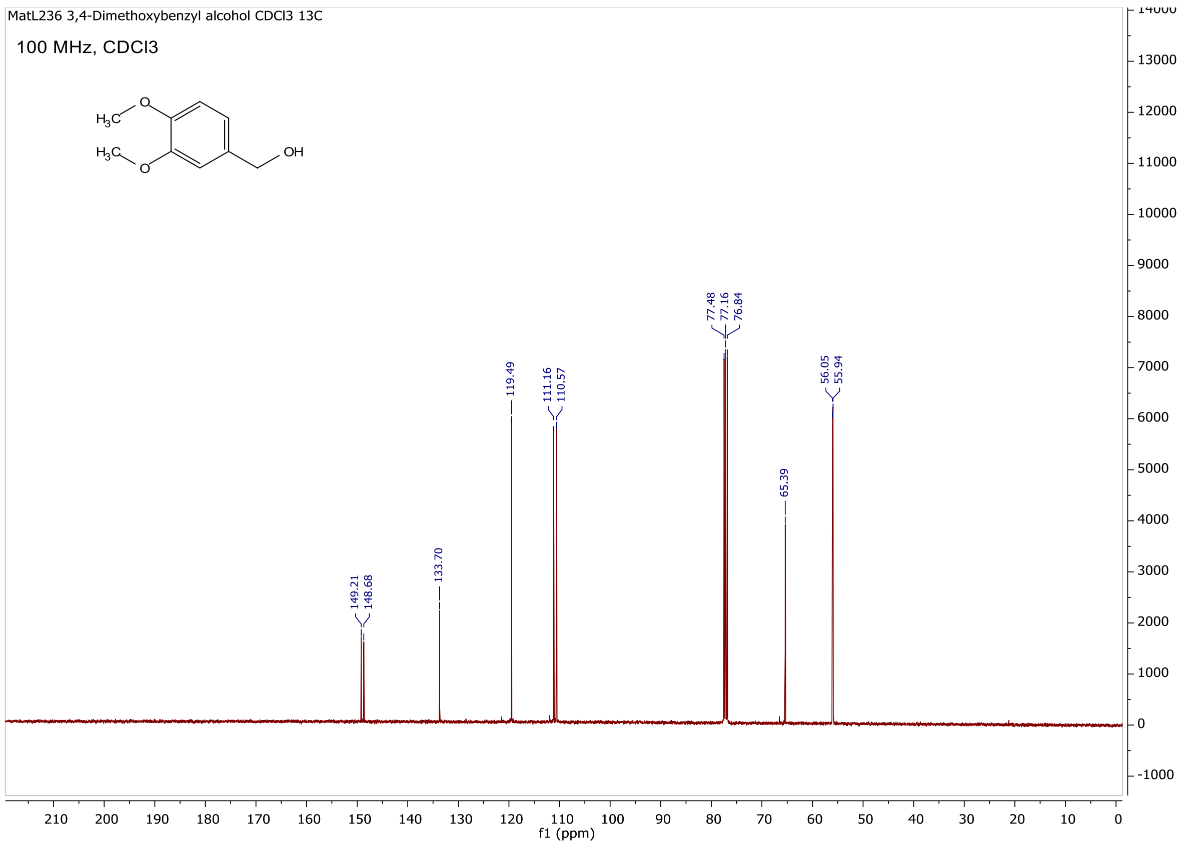

## References


1. Radi, M.; Pagano, M.; Franchi, L.; Castagnolo, D.; Schenone, S.; Casaluce, G.; Zamperini, C.; Dreassi, E.; Maga, G.; Samuele, A.; Gonzalo,E.; Clotet, B.; Esté, J.A.; Botta, M. Synthesis, Biological Activity, and ADME Properties of Novel S-DABOs/N-DABOs as HIV Reverse Transcriptase Inhibitors, *ChemMedChem*, **2012**, *7*, 883–896.
2. Verma, F.; Shukla, P.; Bhardiya, S.R.; Singh, M.; Rai, A.; Rai. V.K. Photocatalytic C(sp3)−H Activation towards $\alpha$-methylenation of Ketones using MeOH as 1 C Source Steering Reagent, *Advanced Synthesis & Catalysis*, **2019**, *361*, 1247–1252.
3. Dabral, S.; Engel, J.; Mottweiler, J.; Spoehrle, S.S.M.; Lahive, C.W.; Bolm, C. Mechanistic studies of base-catalysed lignin depolymerisation in dimethyl carbonate, *Green Chemistry*, **2018**, *20*, 170–182.
4. Štambaský, J.; Malkov, A.V.; Kočovský, P. Synthesis of Enantiopure 1-Arylprop-2-en-1-ols and Their tert-Butyl Carbonates, *The Journal of Organic Chemistry*, **2008**, *73*, 9148–9150.
5. Swain, N.A.; Brown, R.C.D.; Bruton,G. A Versatile Stereoselective Synthesis of endo,exo-Furofuranones: Application to the Enantioselective Synthesis of Furofuran Lignans, *The Journal of Organic Chemistry*, **2004**, *69*, 122–129.
6. Miles-Barrett,D.M.; Neal, A.R.; Hand, C.; Montgomery, J.R.D.; Panovic, I.; Ojo, O.S.; Lancefield, C.S.; Cordes, D.B.; Slawin, A.M.Z.; Lebl,T.; Westwood, N.J. The synthesis and analysis of lignin-bound Hibbert ketone structures in technical lignins, *Organic & Biomolecular Chemistry*, **2016**, *14*, 10023–10030.
7. Abdo, K.M.; Cunningham, M.L.; Snell, M.L.; Herbert, R.A.; Travlos, G.S.; Eldridge, S.R.; Bucher, J.R. 14-Week toxicity and cell proliferation of methyleugenol administered by gavage to F344 rats and B6C3F1 mice, *Food Chem. Toxicol.*, **2001**, *39*, 303–316.
8. Li, S.; Lundquist, K. Reactions of Lignin-related Cinnamaldehydes and Cinnamyl Alcohols with Borane and Sodium Tetrahydridoborate, *Acta Chemica Scandinavica*, **1995**, *49*, 64–67.
9. Sadik, G.; Islam, R.; Rahman, M.M.; Khondkar, P.; Rashid, M.A.; Sarker, S.D. Antimicrobial and cytotoxic constituents of Loranthus globosus, *Fitoterapia*, **2003**, *74*, 308–311.
10. Banerjee,B.; Roy,S.C. Concise Enantioselective Synthesis of Furan Lignans (-)-Dihydrosesamin and (-)-Acuminatin and Furofuran Lignans (-)-Sesamin and (-)-Methyl Piperitol by Radical Cyclization of Epoxides, *Synthesis*, **2005**, 2913–2919.
11. Posner, G.H.; Maxwell, J.P.; Kahraman, M. Mild, Fast, and Stereoselective Epoxide Opening by Ketone Enolate Anions. Application to Synthesis of the Norlignan Curculigine, *The Journal of Organic Chemistry*, **2003**, *68*, 3049–3054.
12. Sawama, Y.; Masuda, M.; Asai, S.; Goto, R.; Nagata, S.; Nishimura, S.; Monguchi, Y.; Sajiki, H. $FeCl_3$-Catalyzed Self-Cleaving Deprotection of Methoxyphenylmethyl-Protected Alcohols, *Organic Letters*, **2015**, *17*, 434–437.
13. Xu, K.; Liu, H.; Liu, D.; Sheng, C.; Shen, J.; Zhang, W. Synthesis of (+)-salvianolic acid A from sodium Danshensu, *Tetrahedron*, **2018**, *74*, 5996–6002.
14. Parihar, S.; Kumar, A.; Chaturvedi, A.K.; Sachan, N.K.; Luqman, S.; Changkija, B.; Manohar, M.; Prakash, O.; Chanda, D.; Khan, F.; Chanotiya, C.S.; Shanker, K.; Dwivedi, A.; Konwar, R.; Negi, A.S. Synthesis of combretastatin A4 analogues on steroidal framework and their anti-breast cancer activity, *The Journal of Steroid Biochemistry and Molecular Biology*, **2013**, *137*, 332–344.
15. O'Byrne, A.; Murray, C.; Keegan, D.; Palacio, C.; Evans, P.; Morgan, B.S. The thio-adduct facilitated, enzymatic kinetic resolution of 4-hydroxycyclopentenone and 4-hydroxycyclohexenone, *Organic & Biomolecular Chemistry*, **2010**, *8*, 539–545.
16. Lundquist, K.; Kirk, T.K. De novo synthesis and decomposition of veratryl alcohol by a lignin-degrading basidiomycete, *Phytochemistry*, **1978**, *17*, 1676.
17. Liu,J.; Yang, C.; Zhang, J.; Wu, J.; Chen, Y. A New 5(6→7)abeo-sterol from the twigs of Podocarpus fleuryi, *Natural Product Research*, **2017**, 31, 175–180.
